# Parametricity for Primitive Nested Types

**DOI:** 10.1007/978-3-030-71995-1_17

**Published:** 2021-03-23

**Authors:** Patricia Johann, Enrico Ghiorzi, Daniel Jeffries

**Affiliations:** 8 University of Oxford, Oxford, UK; 9 Sorbonne Université - LIP6, Paris, France; Appalachian State University, Boone, NC USA

## Abstract

This paper considers parametricity and its resulting free theorems for nested data types. Rather than representing nested types via their Church encodings in a higher-kinded or dependently typed extension of System F, we adopt a functional programming perspective and design a Hindley-Milner-style calculus with primitives for constructing nested types directly as fixpoints. Our calculus can express all nested types appearing in the literature, including truly nested types. At the term level, it supports primitive pattern matching, map functions, and fold combinators for nested types. Our main contribution is the construction of a parametric model for our calculus. This is both delicate and challenging: to ensure the existence of semantic fixpoints interpreting nested types, and thus to establish a suitable Identity Extension Lemma for our calculus, our type system must explicitly track functoriality of types, and cocontinuity conditions on the functors interpreting them must be appropriately threaded throughout the model construction. We prove that our model satisfies an appropriate Abstraction Theorem and verifies all standard consequences of parametricity for primitive nested types.

### 1 Introduction

*Algebraic data types* (ADTs), both built-in and user-defined, have long been at the core of functional languages such as Haskell, ML, Agda, Epigram, and Idris. ADTs, such as that of natural numbers, can be unindexed. But they can also be indexed over other types. For example, the ADT of lists (here coded in Agda)

```
data List (A : Set) : Set where
     nil  : List A
     cons : A → List A → List A
```

is indexed over its element type A. The instance of List at index A depends only on itself, and so is independent of List B for any other index B. That is, List, like all other ADTs, defines a *family of inductive types*, one for each index type.

Over time, there has been a notable trend toward data types whose non-regular indexing can capture invariants and other sophisticated properties that can be used for program verification and other applications. A simple example of such a type is given by Bird and Meertens' [4] prototypical nested type

```
data PTree (A : Set) : Set where
     pleaf : A → PTree A
     pnode : PTree (A × A) → PTree A
```

of perfect trees, which can be thought of as constraining lists to have lengths that are powers of 2. The above code makes clear that perfect trees at index type A are defined in terms of perfect trees at index type A × A. This is typical of nested types, one type instance of which can depend on others, so that the entire family of types must actually be defined at once. A nested type thus defines not a family of inductive types, but rather an *inductive family of types*. Nested types include simple nested types, like perfect trees, none of whose recursive occurrences occur below another type constructor; "deep" nested types [18], such as the nested type

```
data PForest (A : Set) : Set where
    fempty : PForest A
    fnode : A → PTree (PForest A) → PForest A
```

of perfect forests, whose recursive occurrences appear below type constructors for other nested types; and truly nested types, such as the nested type

```
data Bush (A : Set) : Set where
    bnil : Bush A
    bcons : A → Bush (Bush A) → Bush A
```

of bushes, whose recursive occurrences appear below their own type constructors.

*Relational parametricity* encodes a powerful notion of type-uniformity, or representation independence, for data types in polymorphic languages. It formalizes the intuition that a polymorphic program must act uniformly on all of its possible type instantiations by requiring that every such program preserves all relations between pairs of types at which it is instantiated. Parametricity was originally put forth by Reynolds [24] for System F [11], the calculus at the core of all polymorphic functional languages. It was later popularized as Wadler's "theorems for free" [27], so called because it can deduce properties of programs in such languages solely from their types, i.e., with no knowledge whatsoever of the text of the programs involved. Most of Wadler's free theorems are consequences of naturality for polymorphic list-processing functions. However, parametricity can also derive results that go beyond just naturality, such as correctness for ADTs of the program optimization known as *short cut fusion* [10,14].

But what about nested types? Does parametricity still hold if such types are added to polymorphic calculi? More practically, can we justifiably reason type-independently about (functions over) nested types in functional languages?

Type-independent reasoning about ADTs in functional languages is usually justified by first representing ADTs by their Church encodings, and then reasoning type-independently about these encodings. This is typically justified by constructing a parametric model — i.e, a model in which polymorphic functions preserve relations *á la* Reynolds — for a suitable fragment of System F, demonstrating that an initial algebra exists for the positive type constructor corresponding to the functor underlying an ADT of interest, and showing that each such initial algebra is suitably isomorphic to its corresponding Church encoding. In fact, this isomorphism of initial algebras and their Church encodings is one of the "litmus tests" for the goodness of a parametric model.

This approach works well for ADTs, which are always fixpoints of *first-order* functors, and whose Church encodings, which involve quantification over only type variables, are always expressible in System F. For example, $\mathtt{List}\,\mathtt{A}$ is the fixpoint of the first-order functor $F\,X = 1 + A \times X$ and has Church encoding $\forall \alpha.\, \alpha \to (\mathtt{A} \to \alpha \to \alpha) \to \alpha$. But despite Cardelli's [7] claim that "virtually any basic type of interest can be encoded within $F_2$" — i.e., within System

F — non-ADT nested types cannot. Not even our prototypical nested type of perfect trees has a Church encoding expressible in System F! Indeed, `PTree A` cannot be represented as the fixpoint of any *first-order* functor. However, it can be seen as the instance at index `A` of the fixpoint of the *higher-order* functor $H\,F\,A = (A \to F\,A) \to (F\,(A \times A) \to F\,A) \to F\,A$. It thus has Church encoding $\forall f.\,(\forall \alpha.\,\alpha \to f\alpha) \to (\forall \alpha.\,f(\alpha \times \alpha) \to f\alpha) \to \forall \alpha.\,f\alpha$, which requires quantification at the higher kind $* \to *$ for $f$. A similar situation obtains for any (non-ADT) nested type. Unfortunately, higher-kinded quantification is not available in System F, so if we want to reason type-independently about nested types in a language based on it we have only two options: *i*) move to an extension of System F, such as the higher-kinded calculus $F_\omega$ or a dependent type theory, and reason via their Church encodings in a known parametric model for that extension, or *ii*) add nested types to System F as primitives — i.e., as primitive type-level fixpoints — and construct a parametric model for the result.

Since the type systems of $F_\omega$ and dependent type theories are designed to extend System F with far more than non-ADT data types, it seems like serious overkill to pass to their parametric models to reason about nested types in System F. Indeed, such calculi support fundamentally new features that add complexity to their models that is entirely unnecessary for reasoning about nested types. This paper therefore pursues the second option above. We first design a Hindley-Milner-style calculus supporting primitive nested types, together with primitive types of natural transformations representing morphisms between them. Our calculus can express all nested types appearing in the literature, including truly nested types. At the term-level, it supports primitive pattern matching, map functions, and fold combinators for nested types.[1] Our main contribution is the construction of a parametric model for our calculus. This is both delicate and challenging. To ensure the existence of semantic fixpoints interpreting nested types, and thus to establish a suitable Identity Extension Lemma, our type system must explicitly track functoriality of types, and cocontinuity conditions on the functors interpreting them must be appropriately threaded throughout the model construction. Our model validates all standard consequences of parametricity in the presence of primitive nested types, including the isomorphism of primitive ADTs and their Church encodings, and correctness of short cut fusion for nested types. The relationship between naturality and parametricity has long been of interest, and our inclusion of a primitive type of natural transformations allows us to clearly delineate those consequences of parametricity that follow from naturality, from those, such as short cut fusion for nested types, that require the full power of parametricity.

[1] We leave incorporating general term-level recursion to future work because, as Pitts [23] reminds us, "it is hard to construct models of both impredicative polymorphism and fixpoint recursion". In fact, as the development in this paper shows, constructing a parametric model even for our predicative calculus with primitive nested types — and even without term-level fixpoints — is already rather involved. On the other hand, our calculus is strongly normalizing, so it perhaps edges us toward the kind of provably total practical programming language proposed in [27].

**Structure of this Paper** We introduce our calculus in Section 2. Its type system is based on the level-2-truncation of the higher-kinded grammar from [17], augmented with a primitive type of natural transformations. (Since [17] contains no term calculus, the issue of parametricity could not even be raised there.) In Section 3 we give set and relational interpretations of our types. Set interpretations are possible precisely because our calculus is predicative — as ensured by our primitive natural transformation types — and [17] guarantees that local finite presentability of Set makes it suitable for interpreting nested types. As is standard in categorical models, types are interpreted as functors from environments interpreting their type variable contexts to sets or relations, as appropriate. To ensure that these functors satisfy the cocontinuity properties needed for the semantic fixpoints interpreting nested types to exist, set environments must map $k$-ary type constructor variables to appropriately cocontinuous $k$-ary functors on sets, relation environments must map $k$-ary type constructor variables to appropriately cocontinuous $k$-ary relation transformers, and these cocontinuity conditions must be threaded through our type interpretations in such a way that an Identity Extension Lemma (Theorem 1) can be proved. Properly propagating the cocontinuity conditions requires considerable care, and Section 4, where it is done, is (apart from tracking functoriality in the calculus so that it is actually possible) where the bulk of the work in constructing our model lies.

In Section 5, we give set and relational interpretations for the terms of our calculus. As usual in categorical models, terms are interpreted as natural transformations from interpretations of their term contexts to interpretations of their types, and these must cohere in what is essentially a fibred way. In Section 6.1 we prove a scheme deriving free theorems that are consequences of naturality of polymorphic functions over nested types. This scheme is very general, and is parameterized over both the data type and the type of the polymorphic function at hand. It has, for example, analogues for nested types of Wadler's map-rearrangement free theorems as instances. In Section 6.2 we prove that our model satisfies an Abstraction Theorem (Theorem 4), which we use to derive other parametricity results that go beyond naturality. We conclude in Section 7.

**Related Work** There is a long line of work on categorical models of parametricity for System F; see, e.g., [3,6,8,9,12,13,20,26]. To our knowledge, all such models treat ADTs via their Church encodings, verifying in the just-constructed parametric model that each ADT is isomorphic to its encoding. This paper draws on this rich tradition of categorical models of parametricity for System F, but modifies them to treat nested types (and thus ADTs) as primitive data types. The only other extensions we know of System F with primitive data types are those in [19,21,22,23,27]. Wadler [27] treats full System F, and sketches parametricity for its extension with lists. Martin and Gibbons [21] outline a semantics for a grammar of primitive nested types similar to that in [17], but treat only polynomial nested types. Unfortunately, the model suggested in [21] is not entirely correct (see [17]), and parametricity is nowhere mentioned. Matthes [19] treats System F with non-polynomial ADTs and nested types, but focuses on expressivity of generalized Mendler iteration for them. He gives no semantics.

In [23], Pitts adds list ADTs to full System F with a term-level fixpoint primitive. Other ADTs are included in [22], but nested types are not expressible in either syntax. Pitts constructs parametric models for his calculi based on operational, rather than categorical, semantics. A benefit of using operational semantics to build parametric models is that it avoids needing to work in a suitable metatheory to accommodate System F's impredicativity. It is well-known that there are no set-based parametric models of System F [25], so parametric models for it and its extensions are often constructed in a syntactic metatheory such as the impredicative Calculus of Inductive Constructions (iCIC). By adding primitive nested types to a Hindley-Milner-style calculus and working in a categorical setting we side-step such metatheoretic distractions. It is important to note that different consequences of parametricity are available in syntactic and semantic metatheories. Consequences of parametricity are possible for both closed and open System F terms in a syntactic metatheory — although not all that can be formulated can be always proved; see, e.g., the end of Section 7 of [4]. By contrast, in a categorical metatheory consequences of parametricity are expressible only for *closed* terms. For this reason, validating the standard consequences of parametricity for closed terms is — going all the way back to Reynolds [24] — all that is required for a model of parametricity to be considered good.

Atkey [2] treats parametricity for arbitrary higher kinds, constructing a parametric model for System $F_\omega$ within iCIC, rather than in a semantic category. His construction is in some ways similar to ours, but he represents (now higher-kinded) data types using Church encodings rather than as primitives. Moreover, the *fmap* functions associated to Atkey's functors must be *given*, presumably by the programmer, together with their underlying type constructors. This absolves him of imposing cocontinuity conditions on his model to ensure that fixpoints of his functors exist, but, unfortunately, he does not indicate which type constructors support *fmap* functions. We suspect explicitly spelling out which types can be interpreted as strictly positive functors would result in a full higher-kinded extension of a calculus akin to that presented here.

### 2 The Calculus

#### 2.1 Types

For each $k \geq 0$, we assume countable sets $\mathbb{T}^k$ of *type constructor variables of arity* $k$ (i.e., of kind $* \to ... \to * \to *$, with $k$ arrows and $k+1$ $*$s in this sequence) and $\mathbb{F}^k$ of *functorial variables of arity* $k$, all mutually disjoint. The sets of all type constructor variables and functorial variables are $\mathbb{T} = \bigcup_{k\geq 0} \mathbb{T}^k$ and $\mathbb{F} = \bigcup_{k\geq 0} \mathbb{F}^k$, respectively, and a *type variable* is any element of $\mathbb{T} \cup \mathbb{F}$. We use lower case Greek letters for type variables, writing $\phi^k$ to indicate that $\phi \in \mathbb{T}^k \cup \mathbb{F}^k$, and omitting the arity indicator $k$ when convenient. Letters from the beginning of the alphabet denote type variables of arity 0, i.e., elements of $\mathbb{T}^0 \cup \mathbb{F}^0$. We write $\overline{\phi}$ for either a set $\{\phi_1, ..., \phi_n\}$ of type constructor variables or a set of functorial variables when the cardinality $n$ of the set is unimportant or clear from context. If $V$ is a set of type variables we write $V, \overline{\phi}$ for $V \cup \overline{\phi}$ when $V \cap \overline{\phi} = \emptyset$. We omit the vector notation for a singleton set, thus writing $\phi$, instead of $\overline{\phi}$, for $\{\phi\}$.

If $\Gamma$ is a finite subset of $\mathbb{T}$, $\Phi$ is a finite subset of $\mathbb{F}$, $\overline{\alpha}$ is a finite subset of $\mathbb{F}^0$ disjoint from $\Phi$, and $\phi^k \in \mathbb{F}^k \setminus \Phi$, then the set $\mathcal{F}$ of well-formed types is given in Definition 1. The notation there entails that type application $\phi F_1 ... F_k$ is allowed only when $\phi$ is a type variable of arity $k$, or $\phi$ is a subexpression of the form $\mu\psi^k.\lambda\alpha_1...\alpha_k.F'$. Moreover, if $\phi$ has arity $k$ then $\phi$ must be applied to exactly $k$ arguments. Accordingly, an overbar indicates a sequence of subexpressions whose length matches the arity of the type applied to it. Requiring that types are always in such *$\eta$-long normal form* avoids having to consider $\beta$-conversion of types. In a subexpression $\mathsf{Nat}^{\overline{\alpha}} F\, G$, the $\mathsf{Nat}$ operator binds all occurrences of the variables in $\overline{\alpha}$ in $F$ and $G$; intuitively, $\mathsf{Nat}^{\overline{\alpha}} F\, G$ represents the type of a natural transformation in $\overline{\alpha}$ from the functor $F$ to the functor $G$. In a subexpression $\mu\phi^k.\lambda\overline{\alpha}.F$, the $\mu$ operator binds all occurrences of the variable $\phi$, and the $\lambda$ operator binds all occurrences of the variables in $\overline{\alpha}$, in the body $F$.

A *type constructor*, or *non-functorial*, *context* is a finite set $\Gamma$ of type constructor variables, and a *functorial context* is a finite set $\Phi$ of functorial variables. In Definition 1, a judgment of the form $\Gamma;\Phi \vdash F$ indicates that the type $F$ is intended to be functorial in the variables in $\Phi$ but not necessarily in those in $\Gamma$.

**Definition 1.** *The formation rules for the set $\mathcal{F}$ of* (well-formed) types *are*

$$\frac{}{\Gamma;\Phi \vdash \mathbb{0}} \qquad \frac{}{\Gamma;\Phi \vdash \mathbb{1}} \qquad \frac{\Gamma;\Phi \vdash F \qquad \Gamma;\Phi \vdash G}{\Gamma;\Phi \vdash F + G} \qquad \frac{\Gamma;\Phi \vdash F \qquad \Gamma;\Phi \vdash G}{\Gamma;\Phi \vdash F \times G}$$

$$\frac{\Gamma;\overline{\alpha^0} \vdash F \qquad \Gamma;\overline{\alpha^0} \vdash G}{\Gamma;\emptyset \vdash \mathsf{Nat}^{\overline{\alpha^0}} F\, G} \qquad \frac{\phi^k \in \Gamma \cup \Phi \qquad \overline{\Gamma;\Phi \vdash F}}{\Gamma;\Phi \vdash \phi^k \overline{F}}$$

$$\frac{\Gamma;\overline{\alpha^0},\phi^k \vdash F \qquad \overline{\Gamma;\Phi \vdash G}}{\Gamma;\Phi \vdash (\mu\phi^k.\lambda\overline{\alpha^0}.\, F)\,\overline{G}}$$

We write $\vdash F$ for $\emptyset;\emptyset \vdash F$. Definition 1 ensures that the expected weakening rules for well-formed types hold (but weakening does not change the contexts in which types can be formed). If $\Gamma;\emptyset \vdash F$ and $\Gamma;\emptyset \vdash G$, then our rules allow formation of $\Gamma;\emptyset \vdash \mathsf{Nat}^{\emptyset} F\, G$, which represents the arrow type $\Gamma \vdash F \to G$ in our calculus. The type $\Gamma;\emptyset \vdash \mathsf{Nat}^{\overline{\alpha}}\, \mathbb{1}\, F$ represents the $\forall$-type $\Gamma;\emptyset \vdash \forall\overline{\alpha}.F$. Some System F types, such as $\forall\alpha.\,(\alpha \to \alpha) \to \alpha$, are not representable in our calculus.

Since the body F of a type $(\mu\phi.\lambda\overline{\alpha}.F)\overline{G}$ can only be functorial in $\phi$ and the variables in $\overline{\alpha}$, the representation of $\mathit{List}\,\alpha$ as the ADT $\mu\beta.\,\mathbb{1} + \alpha \times \beta$ cannot be functorial in $\alpha$. By contrast, if $\mathit{List}\,\alpha$ is represented as the nested type $(\mu\phi.\lambda\beta.\,\mathbb{1} + \beta \times \phi\beta)\,\alpha$ then we can choose $\alpha$ to be a functorial variable or not when forming the type. This observation holds for other ADTs as well; for example, if $\mathit{Tree}\,\alpha\,\gamma = \mu\beta.\alpha + \beta \times \gamma \times \beta$, then $\alpha,\gamma;\emptyset \vdash \mathit{Tree}\,\alpha\,\gamma$ is well-formed, but $\emptyset;\alpha,\gamma \vdash \mathit{Tree}\,\alpha\,\gamma$ is not. It also applies to some non-ADT types, such as $\mathit{GRose}\,\phi\,\alpha = \mu\beta.\mathbb{1} + \alpha \times \phi\beta$, in which $\phi$ and $\alpha$ must both be non-functorial variables. It is in fact possible to allow "extra" 0-ary functorial variables in the body of $\mu$-types (functorial variables of higher arity are the real problem). This would allow the first-order representations of ADTs to be functorial, but doing so requires some changes to the formation rule for $\mu$-types, as well as the delicate threading of some additional conditions throughout our model construction. But since we can always use an ADT's (semantically equivalent) second-order representation when functoriality is needed, disallowing such "extra" variables does not negatively impact the expressivity of our calculus. We therefore pursue the simpler syntax here.

Definition 1 allows well-formed types to be functorial in no variables. Functorial variables can also be demoted to non-functorial status: if $F[\phi :== \psi]$ is the textual replacement of $\phi$ in $F$, then $\Gamma, \psi^k; \Phi \vdash F[\phi^k :== \psi^k]$ is derivable whenever $\Gamma; \Phi, \phi^k \vdash F$ is. In addition to textual replacement, we also have substitution for types. If $\Gamma; \Phi \vdash F$ is a type, if $\Gamma$ and $\Phi$ contain only type variables of arity 0, and if $k = 0$ for every occurrence of $\phi^k$ bound by $\mu$ in $F$, then we say that $F$ is *first-order*; otherwise we say that $F$ is *second-order*. Substitution for first-order types is the usual capture-avoiding textual substitution. We write $F[\alpha := \sigma]$ for the result of substituting $\sigma$ for $\alpha$ in $F$, and $F[\alpha_1 := F_1, ..., \alpha_k := F_k]$, or $F[\overline{\alpha := F}]$ when convenient, for $F[\alpha_1 := F_1][\alpha_2 := F_2, ..., \alpha_k := F_k]$. The operation $(\cdot)[\phi :=_{\overline{\alpha}} F]$ of *second-order type substitution along* $\overline{\alpha}$ is defined by induction on types exactly as expected. The only interesting clause is that for type application, which defines $(\psi\overline{G})[\phi :=_{\overline{\alpha}} F]$ to be $F[\overline{\alpha := G[\phi :=_{\overline{\alpha}} F]}]$ if $\psi = \phi$ and $\overline{G[\phi :=_{\overline{\alpha}} F]}$ otherwise. Of course, $(\cdot)[\phi^0 :=_{\emptyset} F]$ coincides with first-order substitution. We omit $\overline{\alpha}$ when convenient, but note that it is not correct to substitute along non-functorial variables. It is not hard to see that if $\Gamma; \Phi, \phi^k \vdash H$ and $\Gamma; \Phi, \overline{\alpha} \vdash F$ with $|\overline{\alpha}| = k$, then $\Gamma; \Phi \vdash H[\phi :=_{\overline{\alpha}} F]$. Similarly, if $\Gamma, \phi^k; \Phi \vdash H$, and if $\Gamma; \overline{\psi}, \overline{\alpha} \vdash F$ with $|\overline{\alpha}| = k$ and $\Phi \cap \overline{\psi} = \emptyset$, then $\Gamma, \overline{\psi}'; \Phi \vdash H[\phi :=_{\overline{\alpha}} F[\overline{\psi :== \psi'}]]$.

### 2.2 Terms

Assume an infinite set $\mathcal{V}$ of term variables disjoint from $\mathbb{T}$ and $\mathbb{F}$. If $\Gamma$ is a type constructor context and $\Phi$ is a functorial context, then a *term context for* $\Gamma$ *and* $\Phi$ is a finite set of bindings of the form $x : F$, where $x \in \mathcal{V}$ and $\Gamma; \Phi \vdash F$. We adopt the above conventions for disjoint unions and vectors in term contexts. If $\Delta$ is a term context for $\Gamma$ and $\Phi$ then the formation rules for the set of *well-formed terms over* $\Delta$ are given in Figure 1. An expression $L_{\overline{\alpha}}x.t$ binds all occurrences of the type variables in $\overline{\alpha}$ in the types of $x$ and $t$, as well as all occurrences of $x$ in $t$. In the rule for $t_{\overline{K}}s$ there is one functorial expression in $\overline{K}$ for every variable in $\overline{\alpha}$. In the rule for $\mathsf{map}_H^{\overline{F},\overline{G}}$ there is one functorial expression in $\overline{F}$ and one functorial expression in $\overline{G}$ for each variable in $\overline{\phi}$. Moreover, for each $\phi^k$ in $\overline{\phi}$ the number of variables in $\overline{\beta}$ in the judgments for functorial expresssions in $\overline{F}$ and $\overline{G}$ is $k$. In the rules for $\mathsf{in}_H$ and $\mathsf{fold}_H^F$, the variables in $\overline{\beta}$ are fresh with respect to $H$, and there is one $\beta$ for every $\alpha$. Substitution for terms is the obvious extension of the usual capture-avoiding textual substitution, and weakening is respected.

The "extra" functorial variables in $\overline{\gamma}$ in the rules for $\mathsf{map}_H^{\overline{F},\overline{G}}$ (i.e., those variables not affected by the substitution of $\phi$) allow us to map polymorphic functions over nested types. Suppose, for example, that we want to map the polymorphic function *flatten* : $\mathsf{Nat}^{\beta}(\mathit{PTree}\,\beta)\,(\mathit{List}\,\beta)$ over lists. The $\mathsf{map}$ term for this is typeable as follows:

$$\frac{\Gamma; \alpha, \gamma \vdash \mathit{List}\,\alpha \qquad \Gamma; \gamma \vdash \mathit{PTree}\,\gamma \qquad \Gamma; \gamma \vdash \mathit{List}\,\gamma}{\Gamma; \emptyset \mid \emptyset \vdash \mathsf{map}_{\mathit{List}\,\alpha}^{\mathit{PTree}\,\gamma, \mathit{List}\,\gamma} : \mathsf{Nat}^{\emptyset}(\mathsf{Nat}^{\gamma}(\mathit{PTree}\,\gamma)\,(\mathit{List}\,\gamma))\,(\mathsf{Nat}^{\gamma}\,(\mathit{List}\,(\mathit{PTree}\,\gamma))\,(\mathit{List}\,(\mathit{List}\,\gamma)))}$$

$$\frac{\Gamma;\Phi \vdash F}{\Gamma;\Phi \mid \Delta, x : F \vdash x : F} \qquad \frac{\Gamma;\Phi \mid \Delta \vdash t : \mathbb{0} \qquad \Gamma;\Phi \vdash F}{\Gamma;\Phi \mid \Delta \vdash \bot_F t : F} \qquad \frac{}{\Gamma;\Phi \mid \Delta \vdash \top : \mathbb{1}}$$

$$\frac{\Gamma;\Phi \mid \Delta \vdash s : F}{\Gamma;\Phi \mid \Delta \vdash \mathsf{inL}\ s : F + G} \qquad \frac{\Gamma;\Phi \mid \Delta \vdash t : G}{\Gamma;\Phi \mid \Delta \vdash \mathsf{inR}\ t : F + G}$$

$$\frac{\Gamma;\Phi \vdash F, G \qquad \Gamma;\Phi \mid \Delta \vdash t : F + G \qquad \Gamma;\Phi \mid \Delta, x : F \vdash l : K \qquad \Gamma;\Phi \mid \Delta, y : G \vdash r : K}{\Gamma;\Phi \mid \Delta \vdash \mathsf{case}\, t\, \mathsf{of}\ \{x \mapsto l;\ y \mapsto r\} : K}$$

$$\frac{\Gamma;\Phi \mid \Delta \vdash s : F \qquad \Gamma;\Phi \mid \Delta \vdash t : G}{\Gamma;\Phi \mid \Delta \vdash (s,t) : F \times G} \qquad \frac{\Gamma;\Phi \mid \Delta \vdash t : F \times G}{\Gamma;\Phi \mid \Delta \vdash \pi_1 t : F} \qquad \frac{\Gamma;\Phi \mid \Delta \vdash t : F \times G}{\Gamma;\Phi \mid \Delta \vdash \pi_2 t : G}$$

$$\frac{\Gamma;\overline{\alpha} \vdash F \qquad \Gamma;\overline{\alpha} \vdash G \qquad \Gamma;\overline{\alpha} \mid \Delta, x : F \vdash t : G}{\Gamma;\emptyset \mid \Delta \vdash L_{\overline{\alpha}} x.t : \mathsf{Nat}^{\overline{\alpha}}\, F\, G}$$

$$\frac{\overline{\Gamma;\Phi \vdash K} \qquad \Gamma;\emptyset \mid \Delta \vdash t : \mathsf{Nat}^{\overline{\alpha}}\, F\, G \qquad \Gamma;\Phi \mid \Delta \vdash s : F[\overline{\alpha := K}]}{\Gamma;\Phi \mid \Delta \vdash t_{\overline{K}} s : G[\overline{\alpha := K}]}$$

$$\frac{\Gamma;\overline{\phi},\overline{\gamma} \vdash H \qquad \overline{\Gamma;\overline{\beta},\overline{\gamma} \vdash F} \qquad \overline{\Gamma;\overline{\beta},\overline{\gamma} \vdash G}}{\Gamma;\emptyset \mid \emptyset \vdash \mathsf{map}_H^{\overline{F},\overline{G}} : \mathsf{Nat}^{\emptyset}\ (\overline{\mathsf{Nat}^{\overline{\beta},\overline{\gamma}}\, F\, G})\ (\mathsf{Nat}^{\overline{\gamma}}\, H[\overline{\phi :=_{\overline{\beta}} F}]\ H[\overline{\phi :=_{\overline{\beta}} G}])}$$

$$\frac{\Gamma;\phi,\overline{\alpha} \vdash H}{\Gamma;\emptyset \mid \emptyset \vdash \mathsf{in}_H : \mathsf{Nat}^{\overline{\beta}} H[\phi :=_{\overline{\beta}} (\mu\phi.\lambda\overline{\alpha}.H)\overline{\beta}][\overline{\alpha := \beta}]\ (\mu\phi.\lambda\overline{\alpha}.H)\overline{\beta}}$$

$$\frac{\Gamma;\phi,\overline{\alpha} \vdash H \qquad \Gamma;\overline{\beta} \vdash F}{\Gamma;\emptyset \mid \emptyset \vdash \mathsf{fold}_H^F : \mathsf{Nat}^{\emptyset}\ (\mathsf{Nat}^{\overline{\beta}}\, H[\phi :=_{\overline{\beta}} F][\overline{\alpha := \beta}]\, F)\ (\mathsf{Nat}^{\overline{\beta}}\, (\mu\phi.\lambda\overline{\alpha}.H)\overline{\beta}\, F)}$$

**Fig. 1.** Well-formed terms

However, this derivation would not possible without the "extra" variable $\gamma$.

Our calculus is expressive enough to define, e.g., a function *reversePTree* : $\mathsf{Nat}^{\alpha}\ (PTree\,\alpha)(PTree\,\alpha)$ that reverses the order of the leaves in a perfect tree. It maps the perfect tree $((1,2),(3,4))$ to $((4,3),(2,1))$. Unfortunately, we cannot define recursive functions — such as a concatenation function for perfect trees or a zip function for bushes — that take as inputs a nested type and an argument of another type, both of which are parameterized over the same variable. The fundamental issue is that recursion is expressible only via $\mathsf{fold}$, which produces natural transformations in some variables $\overline{\alpha}$ from $\mu$-types to other functors $F$. The restrictions on $\mathsf{Nat}$-types entail that $F$ cannot itself be a $\mathsf{Nat}$-type containing $\overline{\alpha}$, so, e.g., $\mathsf{Nat}^{\alpha}\ (PTree\,\alpha)(\mathsf{Nat}^{\emptyset}\ (PTree\,\alpha)(PTree\,(\alpha \times \alpha)))$ is not well-typed. Uncurrying gives $\mathsf{Nat}^{\alpha}\ (PTree\,\alpha \times PTree\,\alpha)(PTree\,(\alpha \times \alpha))$, which is well-typed, but $\mathsf{fold}$ cannot produce a term of this type because $PTree\,\alpha \times PTree\,\alpha$ is not a $\mu$-type. Our calculus can, however, express types of recursive functions that take multiple nested types as arguments, provided they are parameterized over disjoint sets of type variables and the return type of the function is parameterized over only the variables occurring in the type of its final argument. Even for ADTs there is a difference between which folds over them we can type when they are viewed as ADTs (i.e., as fixpoints of first-order functors) versus as proper nested types (i.e., as fixpoints of higher-order functors). This is because, in the return type of $\mathsf{fold}$, the arguments of the $\mu$-type must be variables bound by $\mathsf{Nat}$. For ADTs, the $\mu$-type takes no arguments, making it possible to write recursive functions, such as a concatenation function for lists of type $\alpha; \emptyset \vdash \mathsf{Nat}^{\emptyset}\, (\mu\beta.\mathbb{1}+\alpha\times\beta)\, (\mathsf{Nat}^{\emptyset}(\mu\beta.\mathbb{1}+\alpha\times\beta)\, (\mu\beta.\mathbb{1}+\alpha\times\beta))$. This is not possible for nested types — even when they are semantically equivalent to ADTs.

Interestingly, even some recursive functions of a single proper nested type — e.g., a reverse function for bushes that is a true involution — cannot be expressed as folds because the algebra arguments needed to define them are again recursive functions with types of the same problematic form as the type of, e.g., a zip function for perfect trees. Expressivity of folds for nested types has long been a vexing issue, and this is naturally inherited by our calculus. Adding more expressive recursion combinators — e.g., generalized folds or Mendler iterators — could help, but since this is orthogonal to the issue of parametricity in the presence of primitive nested types we do not consider it further here.

## 3 Interpreting Types

We denote the category of sets and functions by $\mathsf{Set}$. The category $\mathsf{Rel}$ has as objects triples $(A, B, R)$, where $R$ is a relation between sets $A$ and $B$. It has as morphisms from $(A, B, R)$ to $(A', B', R')$ pairs $(f : A \to A', g : B \to B')$ of morphisms in $\mathsf{Set}$ such that $(f a, g b) \in R'$ if $(a, b) \in R$. We may write $R : \mathsf{Rel}(A, B)$ for $(A, B, R)$. If $R : \mathsf{Rel}(A, B)$ we write $\pi_1 R$ and $\pi_2 R$ for the *domain* $A$ of $R$ and the *codomain* $B$ of $R$, respectively, and assume $\pi_1$ and $\pi_2$ are surjective. We write $\mathsf{Eq}_A = (A, A, \{(x, x) \mid x \in A\})$ for the *equality relation* on the set $A$.

The key idea underlying Reynolds' parametricity is to give each type $F(\alpha)$ with one free variable $\alpha$ a *set interpretation* $F_0$ taking sets to sets and a *relational interpretation* $F_1$ taking relations $R : \mathsf{Rel}(A, B)$ to relations $F_1(R) : \mathsf{Rel}(F_0(A), F_0(B))$, and to interpret each term $t(\alpha, x) : F(\alpha)$ with one free term variable $x : G(\alpha)$ as a map $t_0$ associating to each set $A$ a function $t_0(A) : G_0(A) \to F_0(A)$. These interpretations are given inductively on the structures of $F$ and $t$ in such a way that they imply two fundamental theorems. The first is an *Identity Extension Lemma*, which states that $F_1(\mathsf{Eq}_A) = \mathsf{Eq}_{F_0(A)}$, and is the essential property that makes a model relationally parametric rather than just induced by a logical relation. The second is an *Abstraction Theorem*, which states that, for any $R : \mathsf{Rel}(A, B)$, $(t_0(A), t_0(B))$ is a morphism in $\mathsf{Rel}$ from $(G_0(A), G_0(B), G_1(R))$ to $(F_0(A), F_0(B), F_1(R))$. The Identity Extension Lemma is similar to the Abstraction Theorem except that it holds for *all* elements of a type's interpretation, not just those that interpret terms. Similar theorems are required for types and terms with any number of free variables.

The key to proving our Identity Extension Lemma is a familiar "cutting down" of the interpretations of universally quantified types to include only the "parametric" elements; the relevant types here are $\mathsf{Nat}$ types. This requires that the set interpretations of types (Section 3.1) are defined simultaneously with their relational interpretations (Section 3.2). While set interpretations are relatively straightforward, relational interpretations are less so because of the cocontinuity conditions needed to know they are well-defined. We develop these conditions in Sections 3.1 and 3.2. This separates our set and relational interpretations in space, but has no other impact on the mutually inductive definitions.

$$
\begin{aligned}
[\![\Gamma;\Phi \vdash \mathbb{0}]\!]^{\mathsf{Set}}\rho &= 0\\
[\![\Gamma;\Phi \vdash \mathbb{1}]\!]^{\mathsf{Set}}\rho &= 1\\
[\![\Gamma;\emptyset \vdash \mathsf{Nat}^{\overline{\alpha}}\,F\,G]\!]^{\mathsf{Set}}\rho &= \{\eta : \lambda\overline{A}.\,[\![\Gamma;\overline{\alpha} \vdash F]\!]^{\mathsf{Set}}\rho[\overline{\alpha := A}] \Rightarrow \lambda\overline{A}.\,[\![\Gamma;\overline{\alpha} \vdash G]\!]^{\mathsf{Set}}\rho[\overline{\alpha := A}]\\
&\qquad \mid \forall\overline{A},\overline{B} : \mathsf{Set}.\forall\overline{R : \mathsf{Rel}(A,B)}.\\
&\qquad (\eta_{\overline{A}},\eta_{\overline{B}}) : [\![\Gamma;\overline{\alpha} \vdash F]\!]^{\mathsf{Rel}}\mathsf{Eq}_\rho[\overline{\alpha := R}] \to [\![\Gamma;\overline{\alpha} \vdash G]\!]^{\mathsf{Rel}}\mathsf{Eq}_\rho[\overline{\alpha := R}]\}\\
[\![\Gamma;\Phi \vdash \phi\overline{F}]\!]^{\mathsf{Set}}\rho &= (\rho\phi)\,\overline{[\![\Gamma;\Phi \vdash F]\!]^{\mathsf{Set}}\rho}\\
[\![\Gamma;\Phi \vdash F + G]\!]^{\mathsf{Set}}\rho &= [\![\Gamma;\Phi \vdash F]\!]^{\mathsf{Set}}\rho + [\![\Gamma;\Phi \vdash G]\!]^{\mathsf{Set}}\rho\\
[\![\Gamma;\Phi \vdash F \times G]\!]^{\mathsf{Set}}\rho &= [\![\Gamma;\Phi \vdash F]\!]^{\mathsf{Set}}\rho \times [\![\Gamma;\Phi \vdash G]\!]^{\mathsf{Set}}\rho\\
[\![\Gamma;\Phi \vdash (\mu\phi.\lambda\overline{\alpha}.H)\overline{G}]\!]^{\mathsf{Set}}\rho &= (\mu T^{\mathsf{Set}}_{H,\rho})\overline{[\![\Gamma;\Phi \vdash G]\!]^{\mathsf{Set}}\rho}\\
\text{where } T^{\mathsf{Set}}_{H,\rho}\,F &= \lambda\overline{A}.[\![\Gamma;\phi,\overline{\alpha} \vdash H]\!]^{\mathsf{Set}}\rho[\phi := F][\overline{\alpha := A}]\\
\text{and } T^{\mathsf{Set}}_{H,\rho}\,\eta &= \lambda\overline{A}.[\![\Gamma;\phi,\overline{\alpha} \vdash H]\!]^{\mathsf{Set}}id_\rho[\phi := \eta][\overline{\alpha := id_A}]
\end{aligned}
$$

**Fig. 2.** Set interpretation

#### 3.1 Interpreting Types as Sets

We interpret types in our calculus as $\omega$-cocontinuous functors on locally finitely presentable categories [1]. Since functor categories of locally finitely presentable categories are again locally finitely presentable, this ensures that the fixpoints interpreting $\mu$-types in Set and Rel exist, and thus that both the set and relational interpretations of all of the types in Definition 1 are well-defined [17]. To bootstrap this process, we interpret type variables as $\omega$-cocontinuous functors. If $\mathcal{C}$ and $\mathcal{D}$ are locally finitely presentable categories, we write $[\mathcal{C},\mathcal{D}]$ for the category of $\omega$-cocontinuous functors from $\mathcal{C}$ to $\mathcal{D}$.

A *set environment* maps each type variable in $\mathbb{T}^k \cup \mathbb{F}^k$ to an element of $[\mathsf{Set}^k,\mathsf{Set}]$. A morphism $f : \rho \to \rho'$ for set environments $\rho$ and $\rho'$ with $\rho|_{\mathbb{T}} = \rho'|_{\mathbb{T}}$ maps each type constructor variable $\psi^k \in \mathbb{T}$ to the identity natural transformation on $\rho\psi^k = \rho'\psi^k$ and each functorial variable $\phi^k \in \mathbb{F}$ to a natural transformation from the $k$-ary functor $\rho\phi^k$ on Set to the $k$-ary functor $\rho'\phi^k$ on Set. Composition of morphisms on set environments is componentwise, with the identity morphism mapping each one to itself. This gives a category of set environments and morphisms between them, denoted SetEnv. We identify a functor in $[\mathsf{Set}^0,\mathsf{Set}]$ with its value on $*$, and consider a set environment to map a type variable of arity 0 to a set. If $\overline{\alpha} = \{\alpha_1,...,\alpha_k\}$ and $\overline{A} = \{A_1,...,A_k\}$, then we write $\rho[\overline{\alpha := A}]$ for the set environment $\rho'$ such that $\rho'\alpha_i = A_i$ for $i = 1,...,k$ and $\rho'\alpha = \rho\alpha$ if $\alpha \notin \{\alpha_1,...,\alpha_k\}$. If $\rho \in \mathsf{SetEnv}$ we write $\mathsf{Eq}_\rho$ for the relation environment (see Section 3) such that $\mathsf{Eq}_\rho v = \mathsf{Eq}_{\rho v}$ for every type variable $v$. The *set interpretation* $[\![\cdot]\!]^{\mathsf{Set}} : \mathcal{F} \to [\mathsf{SetEnv},\mathsf{Set}]$ is defined in Figure 2. The relational interpretations in the second clause of Figure 2 are given in full in Figure 3.

If $\rho \in \mathsf{SetEnv}$ and $\vdash F$ we write $[\![\vdash F]\!]^{\mathsf{Set}}$ for $[\![\vdash F]\!]^{\mathsf{Set}}\rho$ since the environment is immaterial. The third clause of Figure 2 does indeed define a set: local finite presentability of Set and $\omega$-cocontinuity of $[\![\Gamma;\overline{\alpha} \vdash F]\!]^{\mathsf{Set}}\rho$ ensure that the set of natural transformations $\{\eta : [\![\Gamma;\overline{\alpha} \vdash F]\!]^{\mathsf{Set}}\rho \Rightarrow [\![\Gamma;\overline{\alpha} \vdash G]\!]^{\mathsf{Set}}\rho\}$ (which contains $[\![\Gamma;\emptyset \vdash \mathsf{Nat}^{\overline{\alpha}}\,F\,G]\!]^{\mathsf{Set}}\rho$) is a subset of $\big\{([\![\Gamma;\overline{\alpha} \vdash G]\!]^{\mathsf{Set}}\rho[\overline{\alpha := S}])^{([\![\Gamma;\overline{\alpha}\vdash F]\!]^{\mathsf{Set}}\rho[\overline{\alpha:=S}])}$ $\mid \overline{S} = (S_1,...,S_{|\overline{\alpha}|})$, and $S_i$ is a finite set for $i = 1,...,|\overline{\alpha}|\big\}$. There are countably many tuples $\overline{S}$, each giving a morphism from $[\![\Gamma;\overline{\alpha}\vdash F]\!]^{\mathsf{Set}}\rho[\overline{\alpha:=S}]$ to $[\![\Gamma;\overline{\alpha}\vdash G]\!]^{\mathsf{Set}}\rho[\overline{\alpha:=S}]$, and only $\mathsf{Set}$-many such morphisms since $\mathsf{Set}$ is locally small. In addition, $[\![\Gamma;\emptyset\vdash \mathsf{Nat}^{\overline{\alpha}} F\, G]\!]^{\mathsf{Set}}$ is $\omega$-cocontinuous since it is constant on $\omega$-directed sets. Interpretations of $\mathsf{Nat}$ types ensure that $[\![\Gamma\vdash F\to G]\!]^{\mathsf{Set}}$ and $[\![\Gamma\vdash\forall\overline{\alpha}.F]\!]^{\mathsf{Set}}$ are as expected in parametric models.

To make sense of the last clause in Figure 2, we need to know that, for each $\rho\in\mathsf{SetEnv}$, $T^{\mathsf{Set}}_{H,\rho}$ is an $\omega$-cocontinuous endofunctor on $[\mathsf{Set}^k,\mathsf{Set}]$, and thus admits a fixpoint. Since $T^{\mathsf{Set}}_{H,\rho}$ is defined in terms of $[\![\Gamma;\phi,\overline{\alpha}\vdash H]\!]^{\mathsf{Set}}$, interpretations of types must be such functors, which entails that the actions of set interpretations of types on objects and on morphisms in $\mathsf{SetEnv}$ are intertwined. We know from [17] that, for every $\Gamma;\overline{\alpha}\vdash G$, $[\![\Gamma;\overline{\alpha}\vdash G]\!]^{\mathsf{Set}}$ is actually in $[\mathsf{Set}^k,\mathsf{Set}]$ where $k=|\overline{\alpha}|$, so that, for each $[\![\Gamma;\phi^k,\overline{\alpha}\vdash H]\!]^{\mathsf{Set}}$, the corresponding operator $T^{\mathsf{Set}}_H$ can be extended to a *functor* from $\mathsf{SetEnv}$ to $[[\mathsf{Set}^k,\mathsf{Set}],[\mathsf{Set}^k,\mathsf{Set}]]$. The action of $T^{\mathsf{Set}}_H$ on an object $\rho\in\mathsf{SetEnv}$ is given by the higher-order functor $T^{\mathsf{Set}}_{H,\rho}$, whose actions on objects (functors in $[\mathsf{Set}^k,\mathsf{Set}]$) and morphisms between them are given in Figure 2. Its action on a morphism $f:\rho\to\rho'$ is the higher-order natural transformation $T^{\mathsf{Set}}_{H,f}:T^{\mathsf{Set}}_{H,\rho}\to T^{\mathsf{Set}}_{H,\rho'}$ whose action on $F:[\mathsf{Set}^k,\mathsf{Set}]$ is the natural transformation $T^{\mathsf{Set}}_{H,f}\,F: T^{\mathsf{Set}}_{H,\rho}\,F\to T^{\mathsf{Set}}_{H,\rho'}\,F$ whose component at $\overline{A}$ is $(T^{\mathsf{Set}}_{H,f}\,F)_{\overline{A}}=[\![\Gamma;\phi,\overline{\alpha}\vdash H]\!]^{\mathsf{Set}} f[\phi:=id_F][\overline{\alpha:=id_A}]$. The next definition uses $T^{\mathsf{Set}}_H$ to define the functorial action of set interpretation.

**Definition 2.** *The action of* $[\![\Gamma;\Phi\vdash F]\!]^{\mathsf{Set}}$ *on* $f:\rho\to\rho'$ *in* $\mathsf{SetEnv}$ *is given by:*

- $[\![\Gamma;\Phi\vdash \mathbb{0}]\!]^{\mathsf{Set}} f = id_0$
- $[\![\Gamma;\Phi\vdash \mathbb{1}]\!]^{\mathsf{Set}} f = id_1$
- $[\![\Gamma;\emptyset\vdash \mathsf{Nat}^{\overline{\alpha}} F\, G]\!]^{\mathsf{Set}} f = id_{[\![\Gamma;\emptyset\vdash\mathsf{Nat}^{\overline{\alpha}} F\, G]\!]^{\mathsf{Set}}\rho}$
- $[\![\Gamma;\Phi\vdash \phi\overline{F}]\!]^{\mathsf{Set}} f : [\![\Gamma;\Phi\vdash \phi\overline{F}]\!]^{\mathsf{Set}}\rho \to [\![\Gamma;\Phi\vdash \phi\overline{F}]\!]^{\mathsf{Set}}\rho' = (\rho\phi)\overline{[\![\Gamma;\Phi\vdash F]\!]^{\mathsf{Set}}\rho} \to (\rho'\phi)\overline{[\![\Gamma;\Phi\vdash F]\!]^{\mathsf{Set}}\rho'}$ *is defined by* $[\![\Gamma;\Phi\vdash \phi\overline{F}]\!]^{\mathsf{Set}} f = (f\phi)_{\overline{[\![\Gamma;\Phi\vdash F]\!]^{\mathsf{Set}}\rho'}} \circ (\rho\phi)\overline{[\![\Gamma;\Phi\vdash F]\!]^{\mathsf{Set}} f} = (\rho'\phi)\overline{[\![\Gamma;\Phi\vdash F]\!]^{\mathsf{Set}} f}\circ (f\phi)_{\overline{[\![\Gamma;\Phi\vdash F]\!]^{\mathsf{Set}}\rho}}$. *This holds since* $\rho\phi$ *and* $\rho'\phi$ *are functors and* $f\phi:\rho\phi\to\rho'\phi$ *is a natural transformation.*
- $[\![\Gamma;\Phi\vdash F+G]\!]^{\mathsf{Set}} f$ *is defined by* $[\![\Gamma;\Phi\vdash F+G]\!]^{\mathsf{Set}} f(\mathsf{inL}\; x) = \mathsf{inL}\,([\![\Gamma;\Phi\vdash F]\!]^{\mathsf{Set}} f x)$ *and* $[\![\Gamma;\Phi\vdash F+G]\!]^{\mathsf{Set}} f(\mathsf{inR}\; y) = \mathsf{inR}\,([\![\Gamma;\Phi\vdash G]\!]^{\mathsf{Set}} f y)$
- $[\![\Gamma;\Phi\vdash F\times G]\!]^{\mathsf{Set}} f = [\![\Gamma;\Phi\vdash F]\!]^{\mathsf{Set}} f\times [\![\Gamma;\Phi\vdash G]\!]^{\mathsf{Set}} f$
- $[\![\Gamma;\Phi\vdash (\mu\phi.\lambda\overline{\alpha}.H)\overline{G}]\!]^{\mathsf{Set}} f \;:\; [\![\Gamma;\Phi\vdash (\mu\phi.\lambda\overline{\alpha}.H)\overline{G}]\!]^{\mathsf{Set}}\rho \to [\![\Gamma;\Phi\vdash (\mu\phi.\lambda\overline{\alpha}.H)\overline{G}]\!]^{\mathsf{Set}}\rho' = (\mu T^{\mathsf{Set}}_{H,\rho})\overline{[\![\Gamma;\Phi\vdash G]\!]^{\mathsf{Set}}\rho} \to (\mu T^{\mathsf{Set}}_{H,\rho'})\overline{[\![\Gamma;\Phi\vdash G]\!]^{\mathsf{Set}}\rho'}$ *is defined by* $(\mu T^{\mathsf{Set}}_{H,f})\overline{[\![\Gamma;\Phi\vdash G]\!]^{\mathsf{Set}}\rho'}\circ(\mu T^{\mathsf{Set}}_{H,\rho})\overline{[\![\Gamma;\Phi\vdash G]\!]^{\mathsf{Set}} f} = (\mu T^{\mathsf{Set}}_{H,\rho'})\overline{[\![\Gamma;\Phi\vdash G]\!]^{\mathsf{Set}} f}\circ(\mu T^{\mathsf{Set}}_{H,f})\overline{[\![\Gamma;\Phi\vdash G]\!]^{\mathsf{Set}}\rho}$. *This holds since* $\mu T^{\mathsf{Set}}_{H,\rho}$ *and* $\mu T^{\mathsf{Set}}_{H,\rho'}$ *are functors and* $\mu T^{\mathsf{Set}}_{H,f}:\mu T^{\mathsf{Set}}_{H,\rho}\to\mu T^{\mathsf{Set}}_{H,\rho'}$ *is a natural transformation.*

### 3.2 Interpreting Types as Relations

A *$k$-ary relation transformer* $F$ is a triple $(F^1,F^2,F^*)$, where $F^1,F^2:[\mathsf{Set}^k,\mathsf{Set}]$ and $F^*:[\mathsf{Rel}^k,\mathsf{Rel}]$ are functors, if $\overline{R_i:\mathsf{Rel}(A_i,B_i)}$ for $i=1,...,k$ then $F^*\overline{R}:\mathsf{Rel}(F^1\overline{A},F^2\overline{B})$, and if $\overline{(\alpha_i,\beta_i)\in\mathsf{Hom}_{\mathsf{Rel}}(R_i,S_i)}$ for $i=1,...,k$, then $F^*\overline{(\alpha,\beta)}=$

$(F^1\overline{\alpha}, F^2\overline{\beta})$. We define $F\overline{R}$ to be $F^*\overline{R}$ and $F\overline{(\alpha,\beta)}$ to be $F^*\overline{(\alpha,\beta)}$. The last clause above expands to: if $\overline{(a,b) \in R}$ implies $\overline{(\alpha\, a, \beta\, b) \in S}$ then $(c,d) \in F^*\overline{R}$ implies $(F^1\overline{\alpha}\, c, F^2\overline{\beta}\, d) \in F^*\overline{S}$. We identify a 0-ary relation transformer $(A,B,R)$ with $R : \mathsf{Rel}(A,B)$, and write $\pi_1 F$ for $F^1$ and $\pi_2 F$ for $F^2$. Below we extend these conventions to relation environments in the obvious ways.

The category $RT_k$ of $k$-ary relation transformers is given by the following data: an object of $RT_k$ is a $k$-ary relation transformer; a morphism $\delta : (G^1, G^2, G^*) \to (H^1, H^2, H^*)$ in $RT_k$ is a pair of natural transformations $(\delta^1, \delta^2)$ where $\delta^1 : G^1 \to H^1$, $\delta^2 : G^2 \to H^2$ such that, for all $\overline{R : \mathsf{Rel}(A,B)}$, if $(x,y) \in G^*\overline{R}$ then $(\delta^1_{\overline{A}} x, \delta^2_{\overline{B}} y) \in H^*\overline{R}$; and identity morphisms and composition are inherited from the category of functors on $\mathsf{Set}$. An endofunctor $H$ on $RT_k$ is a triple $H = (H^1, H^2, H^*)$, where $H^1$ and $H^2$ are functors from $[\mathsf{Set}^k, \mathsf{Set}]$ to $[\mathsf{Set}^k, \mathsf{Set}]$; $H^*$ is a functor from $RT_k$ to $[\mathsf{Rel}^k, \mathsf{Rel}]$; for all $\overline{R : \mathsf{Rel}(A,B)}$, $\pi_1((H^*(\delta^1,\delta^2))_{\overline{R}}) = (H^1\delta^1)_{\overline{A}}$ and $\pi_2((H^*(\delta^1,\delta^2))_{\overline{R}}) = (H^2\delta^2)_{\overline{B}}$; the action of $H$ on objects is given by $H\,(F^1, F^2, F^*) = (H^1F^1,\ H^2F^2,\ H^*(F^1, F^2, F^*))$; and the action of $H$ on morphisms is given by $H\,(\delta^1,\delta^2) = (H^1\delta^1, H^2\delta^2)$ for $(\delta^1,\delta^2) : (F^1, F^2, F^*) \to (G^1, G^2, G^*)$. Since applying an endofunctor $H$ to $k$-ary relation transformers and morphisms between them must give $k$-ary relation transformers and morphisms between them, this definition implicitly requires the following three conditions to hold: *i)* $H^*(F^1, F^2, F^*)\overline{R} : \mathsf{Rel}(H^1F^1\overline{A}, H^2F^2\overline{B})$ if $R_1 : \mathsf{Rel}(A_1, B_1), ..., R_k : \mathsf{Rel}(A_k, B_k)$; *ii)* $H^*(F^1, F^2, F^*)\,\overline{(\alpha,\beta)} = (H^1F^1\overline{\alpha}, H^2F^2\overline{\beta})$ if $(\alpha_1,\beta_1) \in \mathsf{Hom}_{\mathsf{Rel}}(R_1, S_1), ..., (\alpha_k,\beta_k) \in \mathsf{Hom}_{\mathsf{Rel}}(R_k, S_k)$; and *iii)* if $(\delta^1,\delta^2) : (F^1, F^2, F^*) \to (G^1, G^2, G^*)$ and $R_1 : \mathsf{Rel}(A_1, B_1), ..., R_k : \mathsf{Rel}(A_k, B_k)$, then $((H^1\delta^1)_{\overline{A}} x, (H^2\delta^2)_{\overline{B}} y) \in H^*(G^1, G^2, G^*)\overline{R}$ if $(x,y) \in H^*(F^1, F^2, F^*)\overline{R}$. Note, however, that this last condition is automatically satisfied because it is implied by the third condition on functors on relation transformers.

If $H$ and $K$ are endofunctors on $RT_k$, then a *natural transformation* $\sigma : H \to K$ is a pair $\sigma = (\sigma^1, \sigma^2)$, where $\sigma^1 : H^1 \to K^1$ and $\sigma^2 : H^2 \to K^2$ are natural transformations between endofunctors on $[\mathsf{Set}^k, \mathsf{Set}]$ and the component of $\sigma$ at $F \in RT_k$ is given by $\sigma_F = (\sigma^1_{F^1}, \sigma^2_{F^2})$. This definition entails that $\sigma^i_{F^i}$ is natural in $F^i : [\mathsf{Set}^k, \mathsf{Set}]$, and, for every $F$, both $(\sigma^1_{F^1})_{\overline{A}}$ and $(\sigma^2_{F^2})_{\overline{A}}$ are natural in $\overline{A}$. Moreover, since the results of applying $\sigma$ to $k$-ary relation transformers must be morphisms of $k$-ary relation transformers, it implicitly requires that $(\sigma_F)_{\overline{R}} = ((\sigma^1_{F^1})_{\overline{A}}, (\sigma^2_{F^2})_{\overline{B}})$ is a morphism in $\mathsf{Rel}$ for any $k$-tuple of relations $\overline{R : \mathsf{Rel}(A,B)}$, i.e., that if $(x,y) \in H^*F\overline{R}$, then $((\sigma^1_{F^1})_{\overline{A}} x, (\sigma^2_{F^2})_{\overline{B}} y) \in K^*F\overline{R}$.

Critically, we can compute $\omega$-directed colimits in $RT_k$. Indeed, if $\mathcal{D}$ is an $\omega$-directed set then $\varinjlim_{d\in\mathcal{D}}(F^1_d, F^2_d, F^*_d) = (\varinjlim_{d\in\mathcal{D}} F^1_d, \varinjlim_{d\in\mathcal{D}} F^2_d, \varinjlim_{d\in\mathcal{D}} F^*_d)$. We define an endofunctor $T = (T^1, T^2, T^*)$ on $RT_k$ to be *$\omega$-cocontinuous* if $T^1$ and $T^2$ are $\omega$-cocontinuous endofunctors on $[\mathsf{Set}^k, \mathsf{Set}]$ and $T^*$ is an $\omega$-cocontinuous functor from $RT_k$ to $[\mathsf{Rel}^k, \mathsf{Rel}]$, i.e., is in $[RT_k, [\mathsf{Rel}^k, \mathsf{Rel}]]$. Now, for any $k$, any $A : \mathsf{Set}$, and any $R : \mathsf{Rel}(A,B)$, let $K^{\mathsf{Set}}_A$ be the constantly $A$-valued functor from $\mathsf{Set}^k$ to $\mathsf{Set}$ and $K^{\mathsf{Rel}}_R$ be the constantly $R$-valued functor from $\mathsf{Rel}^k$ to $\mathsf{Rel}$. Also let 0 denote the initial object of either $\mathsf{Set}$ or $\mathsf{Rel}$, as appropriate. Observing that, for every $k$, $K^{\mathsf{Set}}_0$ is initial in $[\mathsf{Set}^k, \mathsf{Set}]$, and $K^{\mathsf{Rel}}_0$ is initial in $[\mathsf{Rel}^k, \mathsf{Rel}]$, we have that, for each $k$, $K_0 = (K^{\mathsf{Set}}_0, K^{\mathsf{Set}}_0, K^{\mathsf{Rel}}_0)$ is initial in $RT_k$. Thus, if

$T = (T^1, T^2, T^*) : RT_k \to RT_k$ is an endofunctor on $RT_k$ we can define the relation transformer $\mu T$ to be $\varinjlim_{n \in \mathbb{N}} T^n K_0 = (\mu T^1, \mu T^2, \varinjlim_{n \in \mathbb{N}} (T^n K_0)^*)$. If $T : [RT_k, RT_k]$ then $\mu T$ is a fixpoint for $T$, i.e., $\mu T \cong T(\mu T)$. The isomorphism is given by $(in_1, in_2) : T(\mu T) \to \mu T$ and $(in_1^{-1}, in_2^{-1}) : \mu T \to T(\mu T)$ in $RT_k$. The latter is always a morphism in $RT_k$, but the former need not be if $T$ is not $\omega$-cocontinuous. Since $\mu T$'s third component is the colimit in $[\mathsf{Rel}^k, \mathsf{Rel}]$ of third components of relation transformers, rather than a fixpoint of an endofunctor on $[\mathsf{Rel}^k, \mathsf{Rel}]$, there is an asymmetry between $\mu T$'s first two and third components.

A *relation environment* maps each type variable in $\mathbb{T}^k \cup \mathbb{F}^k$ to a $k$-ary relation transformer. A morphism $f : \rho \to \rho'$ between relation environments $\rho$ and $\rho'$ with $\rho|_{\mathbb{T}} = \rho'|_{\mathbb{T}}$ maps each $\psi^k \in \mathbb{T}$ to the identity morphism on $\rho\psi^k = \rho'\psi^k$ and each $\phi^k \in \mathbb{F}$ to a morphism from the $k$-ary relation transformer $\rho\phi$ to the $k$-ary relation transformer $\rho'\phi$. Composition of morphisms on relation environments is componentwise, with the identity morphism mapping each to itself; this gives a category $\mathsf{RelEnv}$ of relation environments and their morphisms. We identify a 0-ary relation transformer with its codomain, and consider a relation environment to map a type variable of arity 0 to a relation. We write $\rho[\overline{\alpha := R}]$ for the relation environment $\rho'$ such that $\rho'\alpha_i = R_i$ for $i = 1, ..., k$ and $\rho'\alpha = \rho\alpha$ if $\alpha \notin \{\alpha_1, ..., \alpha_k\}$. If $\rho \in \mathsf{RelEnv}$ we write $\pi_1\rho$ and $\pi_2\rho$ for the set environments mapping each type variable $\phi$ to the functors $(\rho\phi)^1$ and $(\rho\phi)^2$, respectively.

For each $k$, an $\omega$-cocontinuous functor $H : [\mathsf{RelEnv}, RT_k]$ is a triple $H = (H^1, H^2, H^*)$, where $H^1, H^2 : [\mathsf{SetEnv}, [\mathsf{Set}^k, \mathsf{Set}]]$; $H^* : [\mathsf{RelEnv}, [\mathsf{Rel}^k, \mathsf{Rel}]]$; for all $\overline{R : \mathsf{Rel}(A, B)}$ and morphisms $f$ in $\mathsf{RelEnv}$, $\pi_1(H^* f\, \overline{R}) = H^1(\pi_1 f)\, \overline{A}$ and $\pi_2(H^* f\, \overline{R}) = H^2(\pi_2 f)\, \overline{B}$; the action of $H$ on $\rho$ in $\mathsf{RelEnv}$ is given by $H\rho = (H^1(\pi_1\rho),\ H^2(\pi_2\rho),\ H^*\rho)$; and the action of $H$ on morphisms $f : \rho \to \rho'$ in $\mathsf{RelEnv}$ is given by $Hf = (H^1(\pi_1 f), H^2(\pi_2 f))$. The last two points above give: *i*) if $\overline{R_i : \mathsf{Rel}(A_i, B_i)}$, $i = 1, ..., k$, then $H^*\rho\, \overline{R} : \mathsf{Rel}(H^1(\pi_1\rho)\, \overline{A}, H^2(\pi_2\rho)\, \overline{B})$; *ii*) if $\overline{(\alpha_i, \beta_i) \in \mathsf{Hom}_{\mathsf{Rel}}(R_i, S_i)}$, $i = 1, ..., k$, then $H^*\rho\, \overline{(\alpha, \beta)} = (H^1(\pi_1\rho)\, \overline{\alpha}, H^2(\pi_2\rho)\, \overline{\beta})$; and *iii*) if $f : \rho \to \rho'$ and $\overline{R_i : \mathsf{Rel}(A_i, B_i)}$, $i = 1, ..., k$, then if $(x, y) \in H^*\rho\, \overline{R}$ then $(H^1(\pi_1 f)\, \overline{A}\, x, H^2(\pi_2 f)\, \overline{B}\, y) \in H^*\rho'\, \overline{R}$.

Computation of $\omega$-directed colimits in $RT_k$ extends componentwise to colimits in $\mathsf{RelEnv}$. Similarly, $\omega$-cocontinuity for endofunctors on $RT_k$ extends to functors from $\mathsf{RelEnv}$ to $RT_k$. Our relational interpretation $[\![\cdot]\!]^{\mathsf{Rel}} : \mathcal{F} \to [\mathsf{RelEnv}, \mathsf{Rel}]$ is given in Figure 3. It ensures that $[\![\Gamma \vdash F \to G]\!]^{\mathsf{Rel}}$ and $[\![\Gamma \vdash \forall\overline{\alpha}.F]\!]^{\mathsf{Rel}}$ are as expected. As for set interpretations, $[\![\Gamma; \emptyset \vdash \mathsf{Nat}^{\overline{\alpha}} F\, G]\!]^{\mathsf{Rel}}$ is $\omega$-cocontinuous because it is constant on $\omega$-directed sets. If $\rho \in \mathsf{RelEnv}$ we write $[\![\vdash F]\!]^{\mathsf{Rel}}$ for $[\![\vdash F]\!]^{\mathsf{Rel}}\rho$. For the last clause in Figure 3 to be well-defined we need $T_{H,\rho}$ to be an $\omega$-cocontinuous endofunctor on $RT$, so that it admits a fixpoint. Since $T_{H,\rho}$ is defined in terms of $[\![\Gamma; \phi^k, \overline{\alpha} \vdash H]\!]^{\mathsf{Rel}}$, this means that relational interpretations of types must be $\omega$-cocontinuous functors from $\mathsf{RelEnv}$ to $RT_0$, which in turn entails that the actions of relational interpretations of types on objects and on morphisms in $\mathsf{RelEnv}$ are intertwined. We know from [17] that, for every $\Gamma; \overline{\alpha} \vdash F$, $[\![\Gamma; \overline{\alpha} \vdash F]\!]^{\mathsf{Rel}}$ is actually in $[\mathsf{Rel}^k, \mathsf{Rel}]$ where $k = |\overline{\alpha}|$. We first define the actions of each of these functors on morphisms between relation environments, and then argue that they are well-defined and have the required properties. To do this, we

$$
\begin{aligned}
[\![\Gamma;\Phi \vdash \mathbb{0}]\!]^{\mathsf{Rel}}\rho &= 0\\
[\![\Gamma;\Phi \vdash \mathbb{1}]\!]^{\mathsf{Rel}}\rho &= 1\\
[\![\Gamma;\emptyset \vdash \mathsf{Nat}^{\overline{\alpha}}\, F\, G]\!]^{\mathsf{Rel}}\rho &= \{\eta : \lambda\overline{R}.\, [\![\Gamma;\overline{\alpha} \vdash F]\!]^{\mathsf{Rel}}\rho[\overline{\alpha := R}] \Rightarrow \lambda\overline{R}.\, [\![\Gamma;\overline{\alpha} \vdash G]\!]^{\mathsf{Rel}}\rho[\overline{\alpha := R}]\}\\
&= \{(t,t') \in [\![\Gamma;\emptyset \vdash \mathsf{Nat}^{\overline{\alpha}}\, F\, G]\!]^{\mathsf{Set}}(\pi_1\rho) \times [\![\Gamma;\emptyset \vdash \mathsf{Nat}^{\overline{\alpha}}\, F\, G]\!]^{\mathsf{Set}}(\pi_2\rho) \mid\\
&\qquad \forall R_1 : \mathsf{Rel}(A_1,B_1) \ldots R_k : \mathsf{Rel}(A_k,B_k).\\
&\qquad\quad (t_{\overline{A}}, t'_{\overline{B}}) \in ([\![\Gamma;\overline{\alpha} \vdash G]\!]^{\mathsf{Rel}}\rho[\overline{\alpha := R}])^{[\![\Gamma;\overline{\alpha}\vdash F]\!]^{\mathsf{Rel}}\rho[\overline{\alpha:=R}]}\}\\
[\![\Gamma;\Phi \vdash \phi\overline{F}]\!]^{\mathsf{Rel}}\rho &= (\rho\phi)\overline{[\![\Gamma;\Phi \vdash F]\!]^{\mathsf{Rel}}\rho}\\
[\![\Gamma;\Phi \vdash F + G]\!]^{\mathsf{Rel}}\rho &= [\![\Gamma;\Phi \vdash F]\!]^{\mathsf{Rel}}\rho + [\![\Gamma;\Phi \vdash G]\!]^{\mathsf{Rel}}\rho\\
[\![\Gamma;\Phi \vdash F \times G]\!]^{\mathsf{Rel}}\rho &= [\![\Gamma;\Phi \vdash F]\!]^{\mathsf{Rel}}\rho \times [\![\Gamma;\Phi \vdash G]\!]^{\mathsf{Rel}}\rho\\
[\![\Gamma;\Phi \vdash (\mu\phi.\lambda\overline{\alpha}.H)\overline{G}]\!]^{\mathsf{Rel}}\rho &= (\mu T_{H,\rho})\overline{[\![\Gamma;\Phi \vdash G]\!]^{\mathsf{Rel}}\rho}\\
\text{where } T_{H,\rho} &= (T^{\mathsf{Set}}_{H,\pi_1\rho}, T^{\mathsf{Set}}_{H,\pi_2\rho}, T^{\mathsf{Rel}}_{H,\rho})\\
\text{and } T^{\mathsf{Rel}}_{H,\rho}\, F &= \lambda\overline{R}.[\![\Gamma;\phi,\overline{\alpha} \vdash H]\!]^{\mathsf{Rel}}\rho[\phi := F][\overline{\alpha := R}]\\
\text{and } T^{\mathsf{Rel}}_{H,\rho}\, \delta &= \lambda\overline{R}.[\![\Gamma;\phi,\overline{\alpha} \vdash H]\!]^{\mathsf{Rel}} id_\rho[\phi := \delta][\overline{\alpha := id_R}]
\end{aligned}
$$

**Fig. 3.** Relational interpretation extend $T_H$ to a *functor* from $\mathsf{RelEnv}$ to $[[\mathsf{Rel}^k, \mathsf{Rel}], [\mathsf{Rel}^k, \mathsf{Rel}]]$. Its action on an object $\rho \in \mathsf{RelEnv}$ is given by the higher-order functor $T_{H,\rho}$ whose actions on objects and morphisms are given in Figure 3. Its action on a morphism $f : \rho \to \rho'$ is the higher-order natural transformation $T_{H,f} : T_{H,\rho} \to T_{H,\rho'}$ whose action on any $F : [\mathsf{Rel}^k, \mathsf{Rel}]$ is the natural transformation $T_{H,f}\, F : T_{H,\rho}\, F \to T_{H,\rho'}\, F$ whose component at $\overline{R}$ is $(T_{H,f}\, F)_{\overline{R}} = [\![\Gamma;\phi,\overline{\alpha} \vdash H]\!]^{\mathsf{Rel}} f[\phi := id_F][\overline{\alpha := id_R}]$.

Using $T_H$, we can define the functorial action of relational interpretation. The action $[\![\Gamma;\Phi \vdash F]\!]^{\mathsf{Rel}} f$ of $[\![\Gamma;\Phi \vdash F]\!]^{\mathsf{Rel}}$ on $f : \rho \to \rho'$ in $\mathsf{RelEnv}$ is given as in Definition 2, except that all interpretations are relational interpretations and all occurrences of $T^{\mathsf{Set}}_{H,f}$ are replaced by $T_{H,f}$. For this definition and Figure 3 to be well-defined we need that, for every $H$, $T_{H,\rho}\, F$ is a relation transformer, and $T_{H,f}\, F : T_{H,\rho}\, F \to T_{H,\rho'}\, F$ is a morphism of relation transformers, whenever $F$ is a relation transformer and $f : \rho \to \rho'$ is in $\mathsf{RelEnv}$. This is immediate from

$$
[\![\Gamma;\Phi \vdash F]\!] = ([\![\Gamma;\Phi \vdash F]\!]^{\mathsf{Set}}, [\![\Gamma;\Phi \vdash F]\!]^{\mathsf{Set}}, [\![\Gamma;\Phi \vdash F]\!]^{\mathsf{Rel}}) \in [\mathsf{RelEnv}, RT_0] \quad (1)
$$

The proof is a straightforward induction on the structure of $F$, using an appropriate result from [17] to deduce $\omega$-cocontinuity of $[\![\Gamma;\Phi \vdash F]\!]$ in each case.

We can prove by simultaneous induction that set and relational interpretations of types respect demotion of functorial variables to non-functorial ones and, for $\mathsf{D} \in \{\mathsf{Set}, \mathsf{Rel}\}$, $[\![\Gamma;\Phi \vdash G[\overline{\alpha := K}]]\!]^{\mathsf{D}}\rho = [\![\Gamma;\Phi,\overline{\alpha} \vdash G]\!]^{\mathsf{D}}\rho[\overline{\alpha := [\![\Gamma;\Phi \vdash K]\!]^{\mathsf{D}}\rho}]$, and $[\![\Gamma;\Phi \vdash G[\overline{\alpha := K}]]\!]^{\mathsf{D}} f = [\![\Gamma;\Phi,\overline{\alpha} \vdash G]\!]^{\mathsf{D}} f[\overline{\alpha := [\![\Gamma;\Phi \vdash K]\!]^{\mathsf{D}} f}]$, and $[\![\Gamma;\Phi \vdash F[\phi := H]]\!]^{\mathsf{D}}\rho = [\![\Gamma;\Phi,\phi \vdash F]\!]^{\mathsf{D}}\rho[\phi := \lambda\overline{A}.\, [\![\Gamma;\Phi,\overline{\alpha} \vdash H]\!]^{\mathsf{D}}\rho[\overline{\alpha := A}]]$, and, finally, $[\![\Gamma;\Phi \vdash F[\phi := H]]\!]^{\mathsf{D}} f = [\![\Gamma;\Phi,\phi \vdash F]\!]^{\mathsf{D}} f[\phi := \lambda\overline{A}.\, [\![\Gamma;\Phi,\overline{\alpha} \vdash H]\!]^{\mathsf{D}} f[\overline{\alpha := id_A}]]$.

### 4 The Identity Extension Lemma

In most treatments of parametricity, equality relations are taken as *given*, either directly as diagonal relations or perhaps via reflexive graphs. By contrast, we give a categorical definition of graph relations for natural transformations and *construct* equality relations as particular such relations. Our definitions specialize to the usual ones for morphisms between sets and equality relations on sets.

The standard definition $(x, y) \in \langle f \rangle$ iff $fx = y$ of the graph $\langle f \rangle$ of a morphism $f : A \to B$ in $\mathsf{Set}$ naturally generalizes to associate to each natural transformation between $k$-ary functors on $\mathsf{Set}$ a $k$-ary relation transformer. Indeed, if $F, G : \mathsf{Set}^k \to \mathsf{Set}$ and $\alpha : F \to G$ is a natural transformation, then the functor $\langle \alpha \rangle^* : \mathsf{Rel}^k \to \mathsf{Rel}$ is defined as follows. Given $R_1 : \mathsf{Rel}(A_1, B_1), ..., R_k : \mathsf{Rel}(A_k, B_k)$, let $\iota_{R_i} : R_i \hookrightarrow A_i \times B_i$, for $i = 1, ..., k$, be the inclusion of $R_i$ as a subset of $A_i \times B_i$, let $h_{\overline{A \times B}}$ be the unique morphism making the left diagram below commute, and let $h_{\overline{R}} : F\overline{R} \to F\overline{A} \times G\overline{B}$ be $h_{\overline{A \times B}} \circ F\overline{\iota_R}$. Further, let $\alpha^\wedge\overline{R}$ be the subobject through which $h_{\overline{R}}$ is factorized by the mono-epi factorization system in $\mathsf{Set}$, as in the right diagram below. Then $\alpha^\wedge\overline{R} : \mathsf{Rel}(F\overline{A}, G\overline{B})$ by construction, so the action of $\langle \alpha \rangle^*$ on objects can be given by $\langle \alpha \rangle^* \overline{(A, B, R)} = (F\overline{A}, G\overline{B}, \iota_{\alpha^\wedge\overline{R}} \alpha^\wedge\overline{R})$. Its action on morphisms is given by $\langle \alpha \rangle^* \overline{(\beta, \beta')} = (F\overline{\beta}, G\overline{\beta}')$.

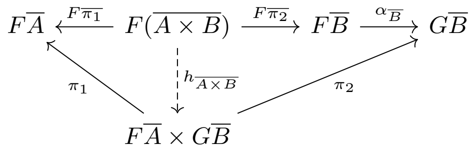

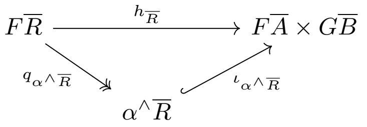

**Lemma 1.** *If* $F, G : [\mathsf{Set}^k, \mathsf{Set}]$*, and if* $\alpha : F \to G$ *is a natural transformation, then the* graph relation transformer for $\alpha$ *defined by* $\langle \alpha \rangle = (F, G, \langle \alpha \rangle^*)$ *is in* $RT_k$*.*

The action of a graph relation transformer on a graph relation can be computed explicitly: if $\alpha : F \to G$ is a morphism in $[\mathsf{Set}^k, \mathsf{Set}]$ and $f_1 : A_1 \to B_1, ..., f_k : A_k \to B_k$, then $\langle \alpha \rangle^* \langle \overline{f} \rangle = \langle G\overline{f} \circ \alpha_{\overline{A}} \rangle = \langle \alpha_{\overline{B}} \circ F\overline{f} \rangle$.

To prove the IEL we also need to know that equality relation transformers preserve equality relations. The *equality relation transformer* on $F : [\mathsf{Set}^k, \mathsf{Set}]$ is $\mathsf{Eq}_F = \langle id_F \rangle = (F, F, \langle id_F \rangle^*)$. The above definition then gives that, for all $\overline{A : \mathsf{Set}}$, $\mathsf{Eq}_F^* \overline{\mathsf{Eq}_A} = \langle id_F \rangle^* \langle id_{\overline{A}} \rangle = \langle F id_{\overline{A}} \circ (id_F)_{\overline{A}} \rangle = \langle id_{F\overline{A}} \circ id_{F\overline{A}} \rangle = \langle id_{F\overline{A}} \rangle = \mathsf{Eq}_{F\overline{A}}$. In addition, if $\rho, \rho' \in \mathsf{SetEnv}$ and $f : \rho \to \rho'$, then the *graph relation environment* $\langle f \rangle$ is defined pointwise by $\langle f \rangle \phi = \langle f \phi \rangle$ for every $\phi$. This entails that $\pi_1 \langle f \rangle = \rho$ and $\pi_2 \langle f \rangle = \rho'$. The *equality relation environment* $\mathsf{Eq}_\rho$ is defined to be $\langle id_\rho \rangle$. Our IEL is thus:

**Theorem 1 (IEL).** *If* $\rho \in \mathsf{SetEnv}$*, then* $[\![\Gamma; \Phi \vdash F]\!]^{\mathsf{Rel}} \mathsf{Eq}_\rho = \mathsf{Eq}_{[\![\Gamma; \Phi \vdash F]\!]^{\mathsf{Set}} \rho}$*.*

The IEL's highly non-trivial proof is by induction on the structure of $F$. Only the $\mathsf{Nat}$, application, and fixpoint cases are non-routine. The latter two explicitly calculate actions of graph relation transformers as above. The fixpoint case also uses that, for every $n \in \mathbb{N}$, the following intermediate results can be proved by simultaneous induction with Theorem 1: for any $H$, $\rho$, $\overline{A}$, and subformula $J$ of $H$, both $T^n_{H, \mathsf{Eq}_\rho} K_0 \, \overline{\mathsf{Eq}_A} = (\mathsf{Eq}_{(T^{\mathsf{Set}}_{H,\rho})^n K_0})^* \overline{\mathsf{Eq}_A}$ and $[\![\Gamma; \Phi, \phi, \overline{\alpha} \vdash J]\!]^{\mathsf{Rel}} \mathsf{Eq}_\rho [\phi := T^n_{H, \mathsf{Eq}_\rho} K_0] \overline{[\alpha := \mathsf{Eq}_A]} = [\![\Gamma; \Phi, \phi, \overline{\alpha} \vdash J]\!]^{\mathsf{Rel}} \mathsf{Eq}_\rho [\phi := \mathsf{Eq}_{(T^{\mathsf{Set}}_{H,\rho})^n K_0}] \overline{[\alpha := \mathsf{Eq}_A]}$ hold.

$$
\begin{array}{ll}
\llbracket \Gamma;\Phi \,|\, \Delta, x : F \vdash x : F \rrbracket^{\mathsf{D}}\rho & = \pi_{|\Delta|+1} \\
\llbracket \Gamma;\emptyset \,|\, \Delta \vdash L_{\overline{\alpha}}x.t : \mathsf{Nat}^{\overline{\alpha}}\, F\, G \rrbracket^{\mathsf{D}}\rho & = \mathsf{curry}(\llbracket \Gamma;\overline{\alpha} \,|\, \Delta, x : F \vdash t : G \rrbracket^{\mathsf{D}}\rho[\overline{\alpha := \_}]) \\
\llbracket \Gamma;\Phi \,|\, \Delta \vdash t_{\overline{K}}s : G[\overline{\alpha := K}] \rrbracket^{\mathsf{D}}\rho & = \mathsf{eval} \circ \langle \lambda d.\, (\llbracket \Gamma;\emptyset \,|\, \Delta \vdash t : \mathsf{Nat}^{\overline{\alpha}}\, F\, G \rrbracket^{\mathsf{D}}\rho\, d)_{\overline{\llbracket \Gamma;\Phi \vdash K \rrbracket^{\mathsf{D}}\rho}}, \\
& \qquad \llbracket \Gamma;\Phi \,|\, \Delta \vdash s : F[\overline{\alpha := K}] \rrbracket^{\mathsf{D}}\rho \rangle \\
\llbracket \Gamma;\Phi \,|\, \Delta \vdash \bot_F t : F \rrbracket^{\mathsf{D}}\rho & = !^{0}_{\llbracket \Gamma;\Phi \vdash F \rrbracket^{\mathsf{D}}\rho} \circ \llbracket \Gamma;\Phi \,|\, \Delta \vdash t : \mathbb{0} \rrbracket^{\mathsf{D}}\rho, \text{ where} \\
& \quad !^{0}_{\llbracket \Gamma;\Phi \vdash F \rrbracket^{\mathsf{D}}\rho} \text{ is the unique morphism from } 0 \\
& \quad \text{to } \llbracket \Gamma;\Phi \vdash F \rrbracket^{\mathsf{D}}\rho \\
\llbracket \Gamma;\Phi \,|\, \Delta \vdash \top : \mathbb{1} \rrbracket^{\mathsf{D}}\rho & = !_{1}^{\llbracket \Gamma;\Phi \vdash \Delta \rrbracket^{\mathsf{D}}\rho}, \text{ where } !_{1}^{\llbracket \Gamma;\Phi \vdash \Delta \rrbracket^{\mathsf{D}}\rho} \\
& \quad \text{is the unique morphism from } \llbracket \Gamma;\Phi \vdash \Delta \rrbracket^{\mathsf{D}}\rho \text{ to } 1 \\
\llbracket \Gamma;\Phi \,|\, \Delta \vdash (s,t) : F \times G \rrbracket^{\mathsf{D}}\rho & = \llbracket \Gamma;\Phi \,|\, \Delta \vdash s : F \rrbracket^{\mathsf{D}}\rho \times \llbracket \Gamma;\Phi \,|\, \Delta \vdash t : G \rrbracket^{\mathsf{D}}\rho \\
\llbracket \Gamma;\Phi \,|\, \Delta \vdash \pi_1 t : F \rrbracket^{\mathsf{D}}\rho & = \pi_1 \circ \llbracket \Gamma;\Phi \,|\, \Delta \vdash t : F \times G \rrbracket^{\mathsf{D}}\rho \\
\llbracket \Gamma;\Phi \,|\, \Delta \vdash \pi_2 t : G \rrbracket^{\mathsf{D}}\rho & = \pi_2 \circ \llbracket \Gamma;\Phi \,|\, \Delta \vdash t : F \times G \rrbracket^{\mathsf{D}}\rho \\
\llbracket \Gamma;\Phi \mid \Delta \vdash \mathsf{case}\, t\, \mathsf{of}\, \{x \mapsto l;\, y \mapsto r\} : K \rrbracket^{\mathsf{D}}\rho & = \mathsf{eval} \circ \langle \mathsf{curry}\, [\llbracket \Gamma;\Phi \,|\, \Delta, x : F \vdash l : K \rrbracket^{\mathsf{D}}\rho, \\
& \qquad\quad \llbracket \Gamma;\Phi \,|\, \Delta, y : G \vdash r : K \rrbracket^{\mathsf{D}}\rho], \\
& \qquad \llbracket \Gamma;\Phi \,|\, \Delta \vdash t : F + G \rrbracket^{\mathsf{D}}\rho \rangle \\
\llbracket \Gamma;\Phi \,|\, \Delta \vdash \mathsf{inL}\ s : F + G \rrbracket^{\mathsf{D}}\rho & = \mathsf{inL} \circ \llbracket \Gamma;\Phi \,|\, \Delta \vdash s : F \rrbracket^{\mathsf{D}}\rho \\
\llbracket \Gamma;\Phi \,|\, \Delta \vdash \mathsf{inR}\ t : F + G \rrbracket^{\mathsf{D}}\rho & = \mathsf{inR} \circ \llbracket \Gamma;\Phi \,|\, \Delta \vdash t : G \rrbracket^{\mathsf{D}}\rho \\
\llbracket \Gamma;\emptyset \,|\, \emptyset \vdash \mathsf{map}_H^{\overline{F},\overline{G}} : \mathsf{Nat}^{\emptyset}(\mathsf{Nat}^{\overline{\beta},\overline{\gamma}} F\, G) & = \lambda d\, \overline{\eta}\, \overline{C}.\, \llbracket \Gamma; \overline{\phi}, \overline{\gamma} \vdash H \rrbracket^{\mathsf{D}} id_{\rho[\overline{\gamma := C}]}[\overline{\phi := \lambda \overline{B}.\eta_{\overline{B}\,\overline{C}}}] \\
\qquad (\mathsf{Nat}^{\overline{\gamma}}\, H[\overline{\phi :=_{\overline{\beta}} F}]\, H[\overline{\phi :=_{\overline{\beta}} G}])\rrbracket^{\mathsf{D}}\rho & \\
\llbracket \Gamma;\emptyset \,|\, \emptyset \vdash \mathsf{in}_H : Nat^{\overline{\beta}}\, H[\phi := (\mu\phi.\lambda\overline{\alpha}.H)\overline{\beta}][\overline{\alpha := \beta}] & = \lambda d.\, in_{T^X_{H,\rho}} \quad \text{where } X \text{ is } \mathsf{Set} \text{ when} \\
\qquad (\mu\phi.\lambda\overline{\alpha}.H)\overline{\beta}\rrbracket^{\mathsf{D}}\rho & \qquad \mathsf{D} = \mathsf{Set} \text{ and not present when } \mathsf{D} = \mathsf{Rel} \\
\llbracket \Gamma;\emptyset \,|\, \emptyset \vdash \mathsf{fold}_H^{F} : \mathsf{Nat}^{\emptyset}\, (\mathsf{Nat}^{\overline{\beta}}\, H[\phi :=_{\overline{\beta}} F][\overline{\alpha := \beta}]\, F) & = \lambda d.\, fold_{T^X_{H,\rho}} \\
\qquad (\mathsf{Nat}^{\overline{\beta}}\, (\mu\phi.\lambda\overline{\alpha}.H)\overline{\beta}\ F)\rrbracket^{\mathsf{D}}\rho & \qquad \text{where } X \text{ is as above}
\end{array}
$$

**Fig. 4.** Term semantics

The case of the proof when $F$ and $J$ are both $\mu$-types makes clear that if functorial variables of arity greater than 0 were allowed to appear in the bodies of $\mu$-types, then the IEL would fail.

With the IEL in hand we can prove a Graph Lemma for our setting:

**Lemma 2.** *If* $\rho, \rho' \in \mathsf{SetEnv}$ *and* $f : \rho \to \rho'$ *then*

$$\langle \llbracket \Gamma;\Phi \vdash F \rrbracket^{\mathsf{Set}} f \rangle = \llbracket \Gamma;\Phi \vdash F \rrbracket^{\mathsf{Rel}} \langle f \rangle$$

### 5 Interpreting Terms

If $\Delta = x_1 : F_1, ..., x_n : F_n$ is a term context for $\Gamma$ and $\Phi$, define $\llbracket \Gamma;\Phi \vdash \Delta \rrbracket^{\mathsf{D}} = \llbracket \Gamma;\Phi \vdash F_1 \rrbracket^{\mathsf{D}} \times ... \times \llbracket \Gamma;\Phi \vdash F_n \rrbracket^{\mathsf{D}}$, where $\mathsf{D}$ is $\mathsf{Set}$ or $\mathsf{Rel}$ as appropriate. Then every well-formed term has a set (resp., relational) interpretation as a natural transformation from the set (resp., relational) interpretation of its term context to that of its type. These interpretations, given in Figure 4, respect weakening, so that $\llbracket \Gamma;\Phi \,|\, \Delta, x : F \vdash t : G \rrbracket^{\mathsf{D}}\rho = (\llbracket \Gamma;\Phi \,|\, \Delta \vdash t : G \rrbracket^{\mathsf{D}}\rho) \circ \pi_\Delta$, where $\rho \in \mathsf{SetEnv}$ or $\rho \in \mathsf{RelEnv}$, and $\pi_\Delta$ is the projection $\llbracket \Gamma;\Phi \vdash \Delta, x : F \rrbracket^{\mathsf{D}} \to \llbracket \Gamma;\Phi \vdash \Delta \rrbracket^{\mathsf{D}}$.

The return type for the semantic fold is $\llbracket \Gamma; \overline{\beta} \vdash F \rrbracket^{\mathsf{D}}\rho[\overline{\beta := B}]$. This interpretation gives $\llbracket \Gamma;\emptyset \,|\, \Delta \vdash \lambda x.t : F \to G \rrbracket^{\mathsf{D}}\rho = \mathsf{curry}(\llbracket \Gamma;\emptyset \,|\, \Delta, x : F \vdash t : G \rrbracket^{\mathsf{D}}\rho)$ and $\llbracket \Gamma;\emptyset \,|\, \Delta \vdash st : G \rrbracket^{\mathsf{D}}\rho = \mathsf{eval} \circ \langle \llbracket \Gamma;\emptyset \,|\, \Delta \vdash s : F \to G \rrbracket^{\mathsf{D}}\rho, \llbracket \Gamma;\emptyset \,|\, \Delta \vdash t : F \rrbracket^{\mathsf{D}}\rho \rangle$, so it specializes to the standard interpretations for System F terms. If $t$ is closed, i.e., if $\emptyset;\emptyset \mid \emptyset \vdash t : F$, then we write $\llbracket \vdash t : F \rrbracket^{\mathsf{D}}$ instead of $\llbracket \emptyset;\emptyset \mid \emptyset \vdash t : F \rrbracket^{\mathsf{D}}$. In addition, term interpretation respects substitution for both functorial and non-functorial type variables, as well as term substitution. Direct calculation reveals that interpretations of terms also satisfy $\llbracket \Gamma;\Phi \mid \Delta \vdash (L_{\overline{\alpha}}x.t)_{\overline{K}}s \rrbracket^{\mathsf{D}} =$

$[\![\Gamma;\Phi \mid \Delta \vdash t[\overline{\alpha := K}][x := s]]\!]^{\mathsf{D}}$. Term extensionality for both types and terms — i.e., $[\![\Gamma;\Phi \vdash (L_\alpha x.t)_\alpha \top : F]\!]^{\mathsf{D}} = [\![\Gamma;\Phi \vdash t : F]\!]^{\mathsf{D}}$ and $[\![\Gamma;\Phi \vdash (L_\alpha x.t)_\alpha x : F]\!]^{\mathsf{D}} = [\![\Gamma;\Phi \vdash t : F]\!]^{\mathsf{D}}$ — follow (when both sides of these equations are defined).

## 6 Free Theorems for Nested Types

### 6.1 Consequences of Naturality

Define, for $\Gamma;\overline{\alpha} \vdash F$, the term $id_F$ to be $\Gamma;\emptyset \mid \emptyset \vdash L_{\overline{\alpha}} x.x : \mathsf{Nat}^{\overline{\alpha}} F\, F$ and, for terms $\Gamma;\emptyset \mid \Delta \vdash t : \mathsf{Nat}^{\overline{\alpha}} F\, G$ and $\Gamma;\emptyset \mid \Delta \vdash s : \mathsf{Nat}^{\overline{\alpha}} G\, H$, the *composition* $s \circ t$ of $t$ and $s$ to be $\Gamma;\emptyset \mid \Delta \vdash L_{\overline{\alpha}} x.s_{\overline{\alpha}}(t_{\overline{\alpha}} x) : \mathsf{Nat}^{\overline{\alpha}} F\, H$. Then $[\![\Gamma;\emptyset \mid \emptyset \vdash id_F : \mathsf{Nat}^{\overline{\alpha}} F\, F]\!]^{\mathsf{Set}} \rho * = id_{\lambda \overline{A}.[\![\Gamma;\overline{\alpha} \vdash F]\!]^{\mathsf{Set}} \rho[\overline{\alpha := A}]}$ for any set environment $\rho$ and $[\![\Gamma;\emptyset \mid \Delta \vdash s \circ t : \mathsf{Nat}^{\overline{\alpha}} F\, H]\!]^{\mathsf{Set}} = [\![\Gamma;\emptyset \mid \Delta \vdash s : \mathsf{Nat}^{\overline{\alpha}} G\, H]\!]^{\mathsf{Set}} \circ [\![\Gamma;\emptyset \mid \Delta \vdash t : \mathsf{Nat}^{\overline{\alpha}} F\, G]\!]^{\mathsf{Set}}$. Also, terms of $\mathsf{Nat}$ type behave as natural transformations with respect to their source and target types:

**Theorem 2.** *If* $\Gamma;\emptyset \mid \Delta \vdash s : \mathsf{Nat}^{\overline{\alpha},\overline{\gamma}} F\, G$ *and* $\overline{\Gamma;\emptyset \mid \Delta \vdash t : \mathsf{Nat}^{\overline{\gamma}} K\, H}$, *then*

$$[\![\Gamma;\emptyset \mid \Delta \vdash ((\mathsf{map}_G^{\overline{K},\overline{H}})_\emptyset\, \overline{t}) \circ (L_{\overline{\gamma}} z.s_{\overline{K},\overline{\gamma}} z) : \mathsf{Nat}^{\overline{\gamma}} F[\overline{\alpha := K}]\, G[\overline{\alpha := H}]]\!]^{\mathsf{Set}}$$
$$= [\![\Gamma;\emptyset \mid \Delta \vdash (L_{\overline{\gamma}} z.s_{\overline{H},\overline{\gamma}} z) \circ ((\mathsf{map}_F^{\overline{K},\overline{H}})_\emptyset\, \overline{t}) : \mathsf{Nat}^{\overline{\gamma}} F[\overline{\alpha := K}]\, G[\overline{\alpha := H}]]\!]^{\mathsf{Set}}$$

Theorem 2 gives rise to an entire family of free theorems that are consequences of naturality, and thus do not require the full power of parametricity. In particular, we can prove that the interpretation of every $\mathsf{map}_H$ is a functor, and that $\mathsf{map}$ is itself a higher-order functor. For example, the former property can be stated as: if $\Gamma;\overline{\alpha},\overline{\gamma} \vdash H$, $\overline{\Gamma;\emptyset \mid \Delta \vdash g : \mathsf{Nat}^{\overline{\gamma}} F\, G}$, and $\overline{\Gamma;\emptyset \mid \Delta \vdash f : \mathsf{Nat}^{\overline{\gamma}} G\, K}$, then

$$[\![\Gamma;\emptyset \mid \Delta \vdash (\mathsf{map}_H^{\overline{F},\overline{K}})_\emptyset\, \overline{(f \circ g)} : \mathsf{Nat}^{\overline{\gamma}} H[\overline{\alpha := F}]\, H[\overline{\alpha := K}]]\!]^{\mathsf{Set}}$$
$$= [\![\Gamma;\emptyset \mid \Delta \vdash (\mathsf{map}_H^{\overline{G},\overline{K}})_\emptyset\, \overline{f} \circ (\mathsf{map}_H^{\overline{F},\overline{G}})_\emptyset\, \overline{g} : \mathsf{Nat}^{\overline{\gamma}} H[\overline{\alpha := F}]\, H[\overline{\alpha := K}]]\!]^{\mathsf{Set}}$$

We can also prove the expected properties of $\mathsf{map}$, $\mathsf{in}$, and $\mathsf{fold}$, and their interpretations, e.g., uniqueness and the universal property of the interpretation of $\mathsf{fold}$, and the interpretation of $\mathsf{in}$ is an isomorphism.

### 6.2 The Abstraction Theorem

To get consequences of parametricity that are not merely consequences of naturality, we prove an Abstraction Theorem (Theorem 4). As usual for such theorems, we prove a more general result (Theorem 3) for open terms, and recover our Abstraction Theorem as its special case for closed terms of closed type.

**Theorem 3.** *Every well-formed term* $\Gamma;\Phi \mid \Delta \vdash t : F$ *induces a natural transformation from* $[\![\Gamma;\Phi \vdash \Delta]\!]$ *to* $[\![\Gamma;\Phi \vdash F]\!]$, *i.e., a triple of natural transformations* $([\![\Gamma;\Phi \mid \Delta \vdash t : F]\!]^{\mathsf{Set}}, [\![\Gamma;\Phi \mid \Delta \vdash t : F]\!]^{\mathsf{Set}}, [\![\Gamma;\Phi \mid \Delta \vdash t : F]\!]^{\mathsf{Rel}})$, *where, for* $\mathsf{D} \in \{\mathsf{Set}, \mathsf{Rel}\}$, *and for* $\rho \in \mathsf{SetEnv}$ *or* $\rho \in \mathsf{RelEnv}$ *as appropriate,* $[\![\Gamma;\Phi \mid \Delta \vdash t : F]\!]^{\mathsf{D}} : [\![\Gamma;\Phi \vdash \Delta]\!]^{\mathsf{D}} \to [\![\Gamma;\Phi \vdash F]\!]^{\mathsf{D}}$ *has component* $[\![\Gamma;\Phi \mid \Delta \vdash t : F]\!]^{\mathsf{D}} \rho : [\![\Gamma;\Phi \vdash \Delta]\!]^{\mathsf{D}} \rho \to [\![\Gamma;\Phi \vdash F]\!]^{\mathsf{D}} \rho$ *at* $\rho$. *Moreover, for all* $\rho \in \mathsf{RelEnv}$, *we have* $[\![\Gamma;\Phi \mid \Delta \vdash t : F]\!]^{\mathsf{Rel}} \rho = ([\![\Gamma;\Phi \mid \Delta \vdash t : F]\!]^{\mathsf{Set}}(\pi_1 \rho), [\![\Gamma;\Phi \mid \Delta \vdash t : F]\!]^{\mathsf{Set}}(\pi_2 \rho))$.

The proof is by induction on $t$. It requires showing that set and relational interpretations of term judgments are natural transformations, and that all set interpretations of terms of $\mathsf{Nat}$-types satisfy the appropriate equality preservation conditions from Figure 2. For the interesting cases of abstraction, application, $\mathsf{map}$, $\mathsf{in}$, and $\mathsf{fold}$ terms, propagating the naturality conditions is somewhat involved; the latter two especially require some delicate diagram chasing. That it is possible provides strong evidence that our development is sensible, natural, and at an appropriate level of abstraction.

Using Theorem 3 we can prove that our calculus admits no terms with the type $\mathsf{Nat}^{\alpha}\mathbb{1}\,\alpha$ of the polymorphic bottom, and every closed term $g$ of type $\mathsf{Nat}^{\alpha}\alpha\,\alpha$ denotes the polymorphic identity function. Moreover, an immediate consequence of Theorem 3 is that if $\rho \in \mathsf{RelEnv}$, and $(a, b) \in [\![\Gamma; \Phi \vdash \Delta]\!]^{\mathsf{Rel}}\rho$, then $([\![\Gamma; \Phi \mid \Delta \vdash t : F]\!]^{\mathsf{Set}}(\pi_1\rho)\, a\, , [\![\Gamma; \Phi \mid \Delta \vdash t : F]\!]^{\mathsf{Set}}(\pi_2\rho)\, b) \in [\![\Gamma; \Phi \vdash F]\!]^{\mathsf{Rel}}\rho$. Its instantiation to closed terms of closed type gives

**Theorem 4 (Abstraction Theorem).** $([\![\vdash t : F]\!]^{\mathsf{Set}}, [\![\vdash t : F]\!]^{\mathsf{Set}}) \in [\![\vdash F]\!]^{\mathsf{Rel}}$

Using Theorem 4 we can recover free theorems, such as that for the type of the standard *filter* function for lists, that go beyond mere naturality, and extend them to those nested types for which analogous functions can be defined. In particular, we can extend short cut fusion for lists [10] to nested types, thereby formally proving correctness of the categorically inspired theorem from [16]. As shown there, replacing $\mathbb{1}$ with any type $\emptyset; \alpha \vdash C$ generalizes Theorem 5 to a free theorem whose conclusion is $\mathit{fold}_H\, B\, \circ\, G\, \mu H\, \mathit{in}_H = G\, [\![\emptyset; \alpha \vdash K]\!]^{\mathsf{Set}}\, B$.

**Theorem 5.** *If* $\emptyset; \phi, \alpha \vdash F$, $\emptyset; \alpha \vdash K$, $H : [\mathsf{Set}, \mathsf{Set}] \to [\mathsf{Set}, \mathsf{Set}]$ *is defined by* $H f x = [\![\emptyset; \phi, \alpha \vdash F]\!]^{\mathsf{Set}}[\phi := f][\alpha := x]$, *and* $G = [\![\phi; \emptyset \mid \emptyset \vdash g : \mathsf{Nat}^{\emptyset}\, (\mathsf{Nat}^{\alpha}\, F\, (\phi\alpha))\, (\mathsf{Nat}^{\alpha}\, \mathbb{1}\, (\phi\alpha))]\!]^{\mathsf{Set}}$ *for some* $g$, *then for every* $B \in H[\![\emptyset; \alpha \vdash K]\!]^{\mathsf{Set}} \to [\![\emptyset; \alpha \vdash K]\!]^{\mathsf{Set}}$ *we have* $\mathit{fold}_H\, B\, (G\, \mu H\, \mathit{in}_H) = G\, [\![\emptyset; \alpha \vdash K]\!]^{\mathsf{Set}}\, B$.

### 7 Conclusion and Directions for Future Work

We have constructed a parametric model for a calculus supporting primitive nested types, and used its Abstraction Theorem to derive free theorems for these types. This was not possible before [17] because these types were not previously known to have well-defined interpretations in locally finitely presentable categories (here, $\mathsf{Set}$ and $\mathsf{Rel}$), and, to our knowledge, no term calculus for them existed either. We naturally hope (some appropriate variant of) the construction elaborated here will generalize to more advanced data types. For example, GADTs can be represented using left Kan extensions, and it was shown in [17] that adding a $\mathsf{Lan}$ construct to a calculus such as ours preserves the $\lambda$-cocontinuity needed for the data types it defines to have well-defined interpretations in locally $\lambda$-presentable categories. (Interestingly, $\lambda > \aleph_1$ is required to interpret even common GADTs.) This suggests carrying out our model construction in locally $\lambda$-presentable cartesian closed categories (lpcccs) $\mathcal{C}$ whose categories of (abstract) relations, obtained by pullback as in [13], are also lpcccs and are appropriately fibred over $\mathcal{C}$. Adding term-level fixpoints further requires our semantic categories not just to be locally $\lambda$-presentable, but to support some kind of domain structure as well.

## References

[1] Adámek, J., Rosický, J.: Locally Presentable and Accessible Categories. Cambridge University Press (1994)

[2] Atkey, R.: Relational Parametricity for Higher Kinds. In: Computer Science Logic, pp. 46–61. Schloss Dagstuhl–Leibniz-Zentrum fuer Informatik (2012)

[3] Bainbridge, E. S., Freyd, P. J., Scedrov, A., Scott, P. J.: Functorial Polymorphism. Theoretical Computer Science 70, 35–64 (1990)

[4] Bird, R., Meertens, L.: Nested datatypes. In: Mathematics of Program Construction, pp. 52–67. Springer (1998)

[5] Bird, R., Paterson, R.: Generalised folds for nested datatypes. Formal Aspects of Computing 11, 200–222 (1999)

[6] Birkedal, L., Møgelberg, R. E.: Categorical models for Abadi and Plotkin’s logic for parametricity. Mathematical Structures in Computer Science 15, 709–772 (2005)

[7] Cardelli, L: Type Systems. In: CRC Handbook of Computer Science and Engineering, pp. 2208–2236. CRC Press (1984)

[8] Dunphy, B., Reddy, U.: Parametric Limits. In: Logic in Computer Science, pp. 242–252. IEEE (2004)

[9] Ghani, N., Johann, P., Nordvall Forsberg, F., Orsanigo, F., Revell, T.: Bifibrational Functorial Semantics for Parametric Polymorphism. Electronic Notes in Theoretical Computer Science 319, 165–181. (2015)

[10] Gill, A., Launchbury, J., Peyton Jones, S. L.: A short cut to deforestation. In: Functional Programming Languages and Computer Architecture, Proceedings, pp. 223–232. Association for Computing Machinery (1993)

[11] Girard, J.-Y.: Interprétation fonctionnelle et élimination des coupures de l’arithmétique d’ordre supérieur. PhD thesis, University of Paris (1972)

[12] Hasegawa, R.: Categorical data types in parametric polymorphism. Mathematical Structures in Computer Science 4, 71–109 (1994)

[13] Jacobs, B.: Categorical Logic and Type Theory. Elsevier (1999)

[14] Johann, P.: A Generalization of Short-Cut Fusion and Its Correctness Proof. Higher-Order and Symbolic Computation 15, 273–300 (2002)

[15] Johann, P., Ghani, N.: Foundations for Structured Programming with GADTs. In: Principles of Programming Languages, pp. 297–308. Association for Computing Machinery (2008)

[16] Johann, P., Ghani, N.: Haskell Programming with Nested Types: A Principled Approach Higher-Order and Symbolic Computation 22(2), 155–189 (2010)

[17] Johann, P., Polonsky, A.: Higher-kinded data types: Syntax and Semantics. In: Logic in Computer Science, pp. 1–13. IEEE (2019)

[18] Johann, P., Polonsky, A.: Deep Induction: Induction Rules for (Truly) Nested Types. In: Foundations of Software Science and Computation Structures, pp. 339–358. Springer (2020)

[19] Matthes, R.: Map Fusion for Nested Datatypes in Intensional Type Theory. Science of Computer Programming 76(3), 204–224 (2011)

[20] Ma, Q., Reynolds, J. C.: Types, abstraction, and parametric polymorphism, part 2. In: Mathematical Foundations of Program Semantics, pp. 1–40. Springer-Verlag (1992)

[21] Martin, C., Gibbons, J.: On the semantics of nested datatypes. Information Processing Letters 80(5), 233–238 (2001)

[22] Pitts, A.: Parametric polymorphism, recursive types, and operational equivalence. (1998)

[23] Pitts, A.: Parametric polymorphism and operational equivalence. Mathematical Structures in Computer Science 10, 321–359 (2000)

[24] Reynolds, J. C.: Types, abstraction, and parametric polymorphism. Information Processing 83(1), 513–523 (1983)

[25] Reynolds, J. C.: Polymorphism is not set-theoretic. Semantics of Data Types, 145–156 (1984)

[26] Robinson, E., Rosolini, G.: Reflexive graphs and parametric polymorphism. In: Logic in Computer Science, pp. 364–371. IEEE (1994)

[27] Wadler, P.: Theorems for free!. In: Functional Programming Languages and Computer Architecture, Proceedings, pp. 347–359. Association for Computing Machinery (1989)

